# Interactions between muscle volume and body mass index on brain structure in the UK Biobank

**DOI:** 10.3389/frdem.2024.1456716

**Published:** 2024-09-23

**Authors:** Alicia Lu, Stephanie Than, Richard Beare, Alexandra La Hood, Taya Annabelle Collyer, Velandai Srikanth, Chris Moran

**Affiliations:** ^1^Peninsula Clinical School, School of Translational Medicine, Monash University, Frankston, VIC, Australia; ^2^Department of Geriatric Medicine, Peninsula Health, Mornington, VIC, Australia; ^3^National Centre for Healthy Ageing, Monash University, Frankston, VIC, Australia; ^4^Department of Geriatric Medicine, Western Health, Footscray, VIC, Australia; ^5^Developmental Imaging, Murdoch Children's Research Institute, Parkville, VIC, Australia; ^6^School of Public Health and Preventive Medicine, Monash University, Melbourne, VIC, Australia; ^7^Department of Home, Acute and Community, Alfred Health, Caulfield, VIC, Australia

**Keywords:** skeletal muscle volume, body composition, brain structure, brain volumes, obesity, UK Biobank

## Abstract

**Background:**

Low skeletal muscle volume may increase dementia risk through mechanisms affecting brain structure. However, it is unclear whether this relationship exists outside of sarcopenia and/or varies by other factors. We aimed to study the interplay between skeletal muscle volume and factors, such as age, sex, and body mass index (BMI), in explaining brain structure at midlife in a cohort without sarcopenia.

**Methods:**

We used abdominal and brain magnetic resonance imaging (MRI) data from a population-based cohort enrolled in the UK Biobank. The following measures were derived: thigh fat-free muscle volume (FFMV), total brain volume (TBV), gray matter volume (GMV), white matter volume (WMV), total hippocampal volume (THV), and white matter hyperintensity volume (WMHV). Participants below sex-based grip strength thresholds suggesting probable sarcopenia were excluded. Linear regression analysis was used to study the interaction or mediation effects of age, sex, and BMI on the associations between FFMV and brain volumes.

**Results:**

Data were available for 20,353 participants (median age 64 years, 53% female). We found interactions between thigh FFMV, BMI, and age (all *p* < 0.05). Greater thigh FFMV was associated with better brain volumes in those aged <64 years with normal (TBV: β = 2.0 ml/L, *p* = 0.004; GMV: β = 0.8 ml/L, *p* = 0.04; WMV: β = 1.1 ml/L, *p* = 0.006; WMHV: β = −0.2 ml/L, *p* = 3.7 × 10^−5^) or low BMI (TBV: β = 21.2 ml/L, *p* = 0.003; WMV: β = 13.3 ml/L, *p* = 0.002, WMHV: β = −1.1 ml/L, *p* = 0.04).

**Conclusion:**

Greater thigh muscle volume correlates with better brain volumes at midlife in people without sarcopenia, but this relationship weakens with greater age and BMI. Further study is required to investigate the underlying mechanisms to understand which components of body composition are potentially modifiable risk factors for dementia.

## 1 Introduction

The pursuit of modifiable risk factors for dementia has led to interest in the roles of skeletal muscle and adiposity in brain aging. Both obesity, a clinical syndrome identifiable by higher adiposity, and low skeletal muscle volume have been linked to increased risks of brain aging and dementia (Jo et al., 2022; Singh-Manoux et al., 2012; Tessier et al., 2022; Uchida, 2023; Yu et al., 2021). Although the biological mechanisms remain complex and are not fully understood, skeletal muscle and adipose tissue are recognized to be highly metabolically active in inflammatory and oxidative stress pathways (Gustafson, 2012; Han et al., 2023; Scisciola et al., 2021). Consequently, low skeletal muscle volume and high adiposity may contribute to brain aging via shared mechanisms, such as increased neuroinflammation and insulin resistance.

Both low skeletal muscle volume and higher adiposity are associated with structural imaging biomarkers of brain aging (Gustafson, 2012; Hamer and Batty, 2019; Moran et al., 2024; Yu et al., 2021). However, little is known about whether they are independently associated with brain structure or whether they interact with each other. With growing evidence of molecular crosstalk between skeletal muscle and adipose tissue (Stanford and Goodyear, 2018), it is possible that any associations between skeletal muscle, especially fat-free lean muscle, and brain structure may differ by the degree of adiposity.

The link between poor skeletal muscle health and poor brain health has been well-documented in those with sarcopenia (Chang et al., 2016; Peng et al., 2020), a pathological state defined by low muscle volume and function (Cruz-Jentoft et al., 2019), although the direction of causality is unknown. The relationship between muscle volume and brain health has shown inconsistencies across studies, likely due to varying approaches in muscle volume measurements, small sample sizes, and differences in study cohort characteristics (Kilgour et al., 2013; Hassan et al., 2019; Tou et al., 2021). It is now known that skeletal muscle produces hormones called myokines, which may improve brain health by reducing neuroinflammation and improving insulin signaling in the brain (Rai and Demontis, 2022). This suggests that greater skeletal muscle volume may offer cognitive benefits through myokine secretion, beyond preventing sarcopenia and its associated cognitive decline. The findings of a recent large-scale study support this theory, with greater genetically proxied lean muscle mass linked to reduced Alzheimer's disease risk in a relatively healthy cohort (Daghlas et al., 2023).

Understanding the relationship between body composition and brain health also requires consideration of the impacts of age and sex. Greater age and female sex are generally associated with lower skeletal muscle volume (Janssen et al., 2000; Keller and Engelhardt, 2013) and increased dementia risk (Podcasy and Epperson, 2016). Many studies have attempted to examine the association between skeletal muscle and brain health measures, such as brain structure or cognitive function. However, these studies have either considered age and sex as covariables rather than modifiers or used small sample sizes that lack the statistical power to perform such analyses (Burns et al., 2010; Tessier et al., 2022; Yu et al., 2021). More evidence is needed to understand whether the associations between skeletal muscle and brain health vary by age or sex.

A better understanding of the relationship between skeletal muscle and brain health and how it is modified by markers of obesity, age, and sex will provide valuable insights into their roles in dementia pathogenesis. Additionally, this may allow groups at higher risk of accelerated brain aging to be identified for potential lifestyle interventions. We therefore aimed to examine the cross-sectional association between skeletal muscle volume and brain structure in a large cohort of people in mid-to-later life living in the United Kingdom (UK) without sarcopenia. We further aimed to explore how any associations between skeletal muscle volume and brain structure were altered by age, sex, and degree of obesity.

## 2 Methods

### 2.1 Study participants

The UK Biobank is a prospective cohort study designed to allow investigators to examine the roles of genetic, lifestyle, and environmental factors in mid-to-later life health and disease (Sudlow et al., 2015). From 2006 to 2010, approximately half a million volunteers aged 40–69 years were recruited from 22 assessment centers in the UK. Detailed recruitment procedures can be found at www.ukbiobank.ac.uk. Data collection involved computer-based questionnaires, in-person interviews, physical and cognitive assessments, biological sampling (blood, urine, and saliva), and genetic analysis. In 2014, 100,000 UK Biobank participants were invited to undergo brain, cardiac, and abdominal magnetic resonance imaging (MRI). Only participants with abdominal MRI (which includes fat-free thigh muscle volume), brain MRI, and hand grip strength measures were included in this study. We excluded participants who met sex-specific cut points for weak grip strength (indicating probable sarcopenia) defined as below 27 kg in men and below 16 kg in women by the European Working Group on Sarcopenia in Older People 2 (EWGSOP2) (Dodds et al., 2020).

### 2.2 Clinical and genetic data

We used age and physical measures obtained during MRI attendance. Ethnicity, educational qualification, smoking, and alcohol use were self-reported by the participants. Socioeconomic status was based on the Townsend Deprivation Index. Apolipoprotein E (ApoE) e4 positivity carried at least one ApoE e4 allele. Hand grip strength in the right hand was measured with a Jamar handheld dynamometer. Total metabolic equivalent task (MET) minutes per week was calculated as the sum of time spent on all activities, including walking and moderate and vigorous activity. The following cardiometabolic factors were derived as binary variables (present/absent) based on a combination of self-reported diagnosis, medication use, and International Classification of Diseases, Tenth Revision (ICD-10) codes collected at baseline and imaging assessments: diabetes from a self-reported diagnosis, the use of insulin or other medications for diabetes mellitus, and/or an ICD-10 code for diabetes mellitus; hypertension from a self-reported diagnosis, medications for hypertension and/or an ICD-10 code for hypertension; ischemic heart disease (IHD) and stroke from a self-reported diagnosis and/or an ICD-10 code for IHD and stroke, respectively; dyslipidemia from medications for dyslipidemia and/or an ICD-10 code for dyslipidemia.

### 2.3 Imaging procedures

Abdominal MRI was performed using a Siemens 1.5T MAGNETOM Aera. The full protocol can be accessed at https://biobank.ndph.ox.ac.uk/ukb/ukb/docs/AbdoBodyCompMethod.pdf. Fat and water image volumes were corrected and calibrated using an algorithm with pure adipose tissue as the internal signal reference. The images were merged into composite sets covering the neck to the knees. Atlases with ground truth labels for fat and muscle compartments were registered to the acquired volumes using non-rigid atlas-based registration. Posterior thigh muscles were defined as gluteus, iliacus, adductor, and hamstring muscles on respective sides. Anterior thigh muscles were defined as quadriceps femoris and sartorius. Total thigh fat-free muscle volume (FFMV) was calculated as the total volume of voxels with fat fraction < 50% in both thighs (Borga et al., 2018). Tissue volumes were quantified through an automatic segmentation process, adjusted by a trained operator, and calculated through the integration of the calibrated adipose tissue imaging (West et al., 2016). Exemplar images of the MRI muscle segmentation have been previously published (Thanaj et al., 2023; Linge et al., 2021).

We chose MRI thigh fat-free muscle volume as a surrogate measure of whole-body muscle volume as several studies have demonstrated a strong correlation between thigh skeletal muscle and whole-body skeletal muscle (Lee et al., 2004; Mourtzakis et al., 2008; Weise et al., 2013). Although dual-energy X-ray absorptiometry is cheaper and more readily available, MRI is considered the gold standard for the assessment of muscle volume (Prado and Heymsfield, 2014) and correlates best with cadaveric values (Engstrom et al., 1991; Mitsiopoulos et al., 1998).

Brain MRI images were obtained using a Siemens Skyra 3T system with a standard Siemens 32-channel RF receive head coil. The full protocol can be viewed at https://biobank.ctsu.ox.ac.uk/crystal/crystal/docs/brain_mri.pdf. Imaging-derived phenotypes (IDPs) were generated by an image-processing pipeline developed and run on behalf of the UK Biobank (Alfaro-Almagro et al., 2018). We used the following IDPs derived from T1-weighted and T2-weighted fluid-attenuated inversion recovery scans: total brain volume (TBV), total and regional gray matter volume (GMV), white matter volume (WMV), total hippocampal volume (right + left hippocampus, THV), and white matter hyperintensity volume (WMHV). The UK Biobank brain imaging group conducted normalization of some brain volumes using a structural image evaluation, using normalization, of atrophy: cross-sectional (SIENAX)-style analysis (Smith et al., 2002). TBV, GMV, WMV, and THV were normalized for head size. WMHV was log-transformed, given its skewed distribution. Exemplar images of the MRI brain volumes across different BMI categories are presented in Supplementary Figure 1.

### 2.4 Statistical analyses

Summary statistics were used to describe the sample. We used multivariable linear regression to examine the associations between thigh FFMV and each normalized brain volume measure (TBV, GMV, WMV, THV, and WMHV). In a previously published study using data from the UK Biobank, we identified an interaction between age and sex on TBV, GMV, and WMHV (Than et al., 2021). As such, we first added this age × sex interaction term to univariable models before examining it for additional interactions. Interactions were assessed using a product term. The nature of interactions between the primary variables of interest, FFMV, and BMI, in explaining brain volumes was first explored by stratification for sex, age, and BMI categories. Interactions involving age were explored by stratification into two groups using a cut point at the median age of the sample (i.e., 64 years). For interactions involving BMI, we stratified it into four categories (< 18.5, 18.5 to 24.99, 25 to 29.99, ≥30) based on commonly used clinical cut points (Weir and Jan, 2023). When an interaction involving BMI was identified, we examined each BMI stratum as a reference group to assess whether other BMI strata differed significantly. We used the “simple_slopes” command within the R package “reghelper” to estimate associations within interaction terms (Hughes, 2023). Covariables in fully adjusted models included education, Townsend Deprivation Index (TDI), ethnicity, smoking status, alcohol, cardiometabolic factors (hypertension, diabetes, hypercholesterolemia, ischemic heart disease, stroke), MET minutes of physical activity per week, ApoE e4 carrier status, and handgrip strength.

Additionally, the whole cohort was stratified into two subgroups based on APOE e4 status, with the linear regression analysis repeated within each group to explore potential differences in the associations between thigh FFMV and each brain volume. Interactions between thigh FFMV and BMI were also assessed within each group. ApoE e4 carrier status was excluded from the covariables in the fully adjusted models. All statistical analyses were performed using R version 4.2.1(R Core Team, 2022) in the RStudio computing environment version 2022.07.1 (RStudio Team, 2022).

## 3 Results

Complete thigh muscle and brain volumetric data were available for 20,353 participants after excluding those meeting the criteria for probable sarcopenia (Table 1). The median age was 64 years, and the majority of the participants self-identified as female (53.1%) and white (97.0%). Almost half of the participants had a tertiary degree (47%). The mean BMI was 26.5 ± 4.3 kg/m^2^. The median thigh FFMV was 9.8 L.

**Table 1:** Participant characteristics.

	**Whole group Mean ±SD or median (IQR) or *n* (%)**
*n*	20,353
Age (years)	64 (57–69)
Female sex	10,808 (53.1%)
Townsend deprivation index	−2.7 (from −4.0 to −0.8)
Tertiary education	9,606 (47.2%)
White ethnicity	19,749 (97.0%)
Ever smoked	10,672 (52.4%)
Current or previous alcohol drinker	19,584 (96.2%)
Sleep duration (h)	7 (7–8)
Depressed mood in last 2 weeks	3,146 (15.5%)
Hypertension	7,487 (36.8%)
Diabetes	1,028 (5.1%)
Hypercholesterolemia	4,657 (22.9%)
Ischemic heart disease	1,042 (5.1%)
Stroke	725 (3.6%)
BMI (kg/m^2^)	26.5 ± 4.3
< 18.5	138 (0.7%)
18.5–24.9	8,150 (40.0%)
25–29.9	8,398 (41.3%)
≥30	3,595 (17.7%)
Systolic blood pressure (mm Hg)	139.0 ± 19.2
Diastolic blood pressure (mm Hg)	78.6 ± 10.6
Physical activity	
Low	3,202 (15.7%)
Moderate	7,148 (35.1%)
High	6,862 (33.7%)
Total MET minutes per week	1,716 (815–3,279)
Walking pace	
Slow (< 3 miles/h)	722 (3.6%)
Steady (3–4 miles/h)	9,982 (49.0%)
Brisk (>4 miles/h)	9,520 (46.8%)
Right-hand grip strength (kg)	30.0 (24–40)
Apolipoprotein E e4 carrier	5,485 (26.9%)
Total thigh FFMV (L)	9.8 (8.1–12.2)
Total brain volume (ml)	1,502.7 ± 72.6
Gray matter volume (ml)	796.4 ± 47.8
White matter volume (ml)	706.3 ± 40.8
Total hippocampal volume (ml)	10.0 ± 1.1
Total volume of white matter hyperintensities (ml)	3.4 (1.9–6.8)

*n*, number; BMI, body mass index; FFMV, fat-free muscle volume; IQR, interquartile range; MET; metabolic equivalent of task; SD, standard deviation.

Characteristics of those meeting the criteria for probable sarcopenia can be found in Supplementary Table 1, while characteristics of the ApoE e4 positive and negative subgroups are outlined in Supplementary Table 2.

### 3.1 Thigh muscle volume, body mass index, and brain volumes

Table 2 presents the associations between thigh FFMV and normalized brain volumes. Comparative analysis and linear regression analysis of these associations across different age and BMI groups are presented in Supplementary Tables 3, 4, respectively. In models with TBV, GMV, WMV, THV, and WMHV as outcomes, we identified an interaction between age and sex (for interaction, *p* < 0.001), supporting the use of this product term in regression models.

**Table 2:** Associations and interactions between thigh FFMV, BMI, and normalized brain volumes.

	**Total brain volume β for regression model (95% CI)**	**Gray matter volume β for regression model (95% CI)**	**White matter volume β for regression model (95% CI)**	**Total hippocampal volume β for regression model (95% CI)**	**White matter hyperintensity volume β for regression model (95% CI)**
**Model 1**					
(FFMV)	−2.3 (−2.7, −1.9)^*^	−4.3 (−4.5, −4.0)^*^	1.9 (1.7, 2.2)^*^	−0.10 (−0.1, −0.09)^*^	−0.07 (−0.1, −0.03)^*^
**Model 2**					
(FFMV + age × sex)	−2.7 (−3.3, −2.1)^*^−0.8 (−1.0, −0.6)^*^	−2.8 (−3.1, −2.4)^*^ −0.5 (−0.6, −0.3)^*^	0.04 (−0.3, 0.4) −0.3 (−0.5, −0.2)^*^	−0.06 (−0.07, −0.05)^*^ 0.02 (0.01, 0.02)^*^	0.09 (0.02, 0.2)^*^ 0.03 (0.006, 0.06)^*^
**Model 3**					
(FFMV × BMI + age × sex)	−0.2 (−0.3, −0.2)^*^−0.8 (−1.0, −0.6)^*^	−0.2 (−0.2, −0.1)^*^ −0.5 (−0.6, −0.3)^*^	−0.07 (−0.1, −0.03)^*^−0.3 (−0.5, -0.2)^*^	−0.001 (−0.003, 0.00007) 0.02 (0.01, 0.02)^*^	0.03 (0.02, 0.04)^*^ 0.03 (0.009, 0.06)^*^
**Model 4** (fully adjusted)^a^					
(FFMV × BMI + age × sex)	−0.2 (−0.3, −0.2)^*^−0.8 (−1.1, −0.6)^*^	−0.2 (−0.2, −0.1)^*^ −0.5 (−0.7, −0.4)^*^	−0.08 (−0.1, −0.03)^*^−0.3 (−0.5, −0.2)^*^	−0.002 (−0.003, 0.0003)^*^ 0.02 (0.01, 0.02)^*^	0.03 (0.02, 0.04)^*^ 0.03 (0.005, 0.06)^*^

BMI, Body mass index; CI, confidence interval; FFMV, fat-free muscle volume.

^a^Model includes Townsend deprivation index, education, ethnicity, smoking, alcohol, hypertension, diabetes, hypercholesterolemia, ischemic heart disease, stroke, metabolic equivalent minutes of physical activity per week, Apolipoprotein E ε4 carrier status.

^*^p < 0.05.

#### 3.1.1 Total brain volume

Greater thigh FFMV was associated with smaller TBV (model 1, β = −2.3 ml/L, *p* < 2 × 10^−16^). The association between thigh FFMV and TBV remained when the age × sex interaction was included in the model (model 2, β = −2.7 ml/L, *p* < 2 × 10^−16^). We additionally found an interaction between thigh FFMV and BMI (model 3, β = −0.2, *p* = 2.7 × 10^−10^), which persisted in the fully adjusted model (model 4, β = −0.2, p = 7.4 × 10^−9^).

To explore the nature of the thigh FFMV × BMI interaction given the existing age × sex interaction, we first examined the presence of the FFMV × BMI interaction when stratified by sex in fully adjusted models (Supplementary Table 5). We did not find a thigh FFMV × BMI interaction either in women (*p* = 0.20) or in men (*p* = 0.41).

**Figure 1:**
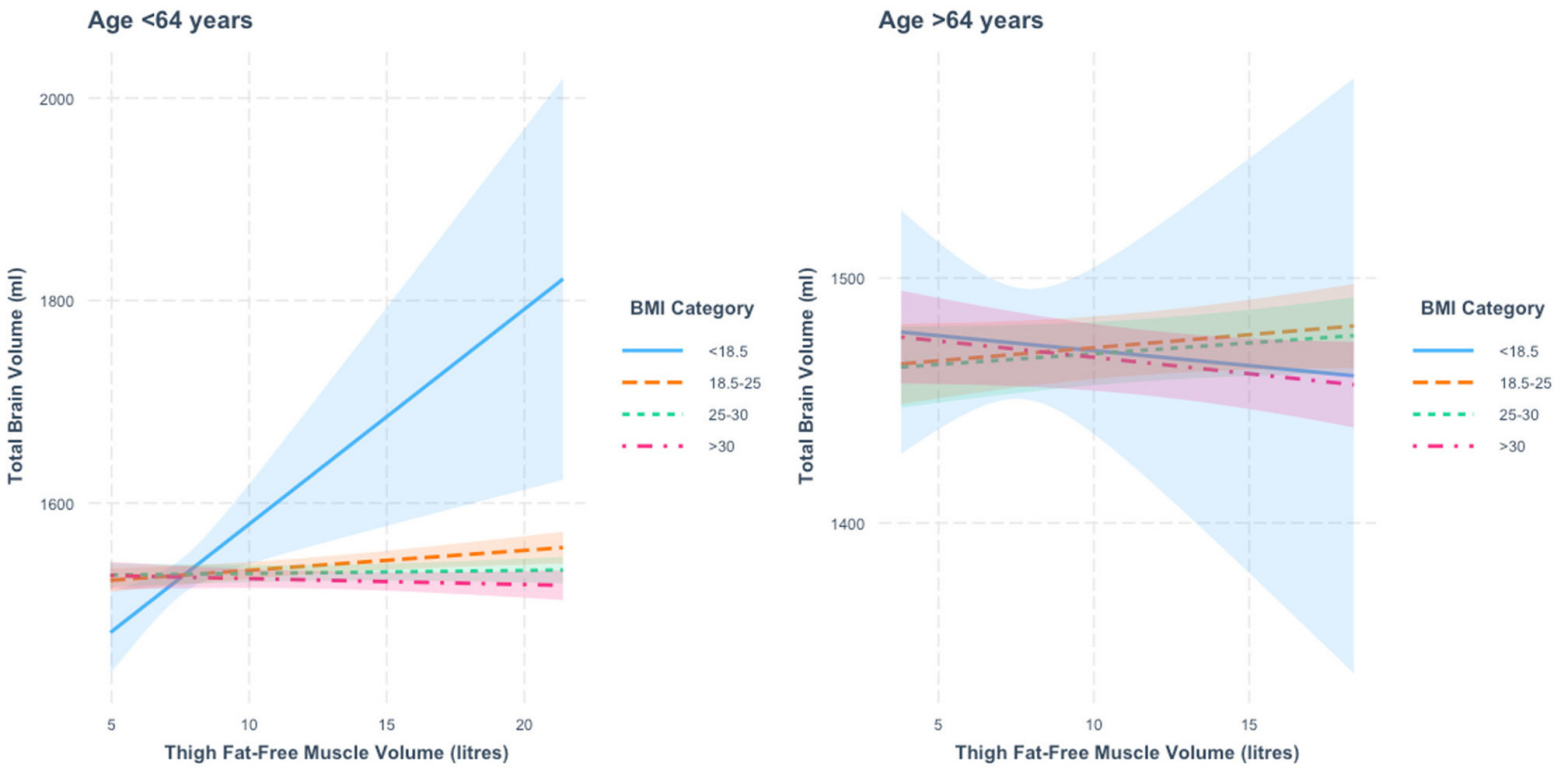
Association between thigh fat-free muscle volume (FFMV) and total brain volume by body mass index (BMI) stratified by age. Shaded areas represent 95% confidence intervals (CIs).

When stratified by age, we found a thigh FFMV × BMI interaction in those below 64 years of age (*p* = 3.3 × 10^−5^). Relative to those with BMI < 18.5, we found that the associations between thigh FFMV and TBV differed in the other three BMI strata (all *p* < 0.05). Figure 1 presents the nature of this interaction. When stratified by BMI, we found an association between greater thigh FFMV and greater TBV in those not obese. The positive association was greatest in those with BMI < 18.5 (β = 21.2 ml/L, *p* = 0.003), followed by those with BMI 18.5–24.9 (β = 2.0 ml/L, *p* = 0.004). No significant association was observed in the overweight and obese categories.

In those 64 years or older, we also found a thigh FFMV × BMI interaction with TBV (*p* = 0.001), with distinct differences across BMI strata. Figure 1 presents this interaction. Specifically, we found that the association between thigh FFMV and TBV was different in those with BMI ≥30 relative to those with BMI 18.5–24.9 (*p* = 0.008) and those with BMI 25–29.9 (*p* = 0.01) (Supplementary Table 3). When analysis of the thigh FFMV–TBV association was stratified by BMI, although not statistically significant, we observed positive associations in those with BMI 18.5–24.9 (β = 1.1 ml/L, *p* = 0.17) and BMI 25–29.9 (β = 0.9 ml/L, *p* = 0.20) and negative associations in the groups with BMI < 18.5 (β = −1.2 ml/L, *p* = 0.83) and BMI >30 (β = −1.3 ml/L, *p* = 0.12). To assist visualization, rescaled graphs, with those with BMI < 18.5 not plotted, are presented in Supplementary Figure 2.

#### 3.1.2 Gray matter volume

Greater thigh FFMV was associated with smaller GMV (model 1, β = −4.3 ml/L, *p* < 2 × 10^−16^). The association between thigh FFMV and GMV remained when the age × sex interaction was included in the model (model 2, β = −2.8 ml/L, *p* < 2 × 10^−16^). We additionally found an interaction between thigh FFMV and BMI on GMV (model 3, β = −0.2, *p* = 3.0 × 10^−13^), which persisted when potential confounders were included (model 4, β = −0.2, *p* = 4.1 × 10^−11^).

To explore the nature of the thigh FFMV × BMI interaction, we first examined for the presence of the interaction when stratified by sex in fully adjusted models (Supplementary Table 5). The thigh FFMV × BMI interaction term was statistically significant in women (*p* = 0.03). However, when stratified by BMI, the associations between thigh FFMV and GMV in all four BMI strata were statistically similar (all *p* > 0.05), regardless of the reference BMI group chosen. There was no thigh FFMV × BMI interaction in men (*p* = 0.06).

**Figure 2:**
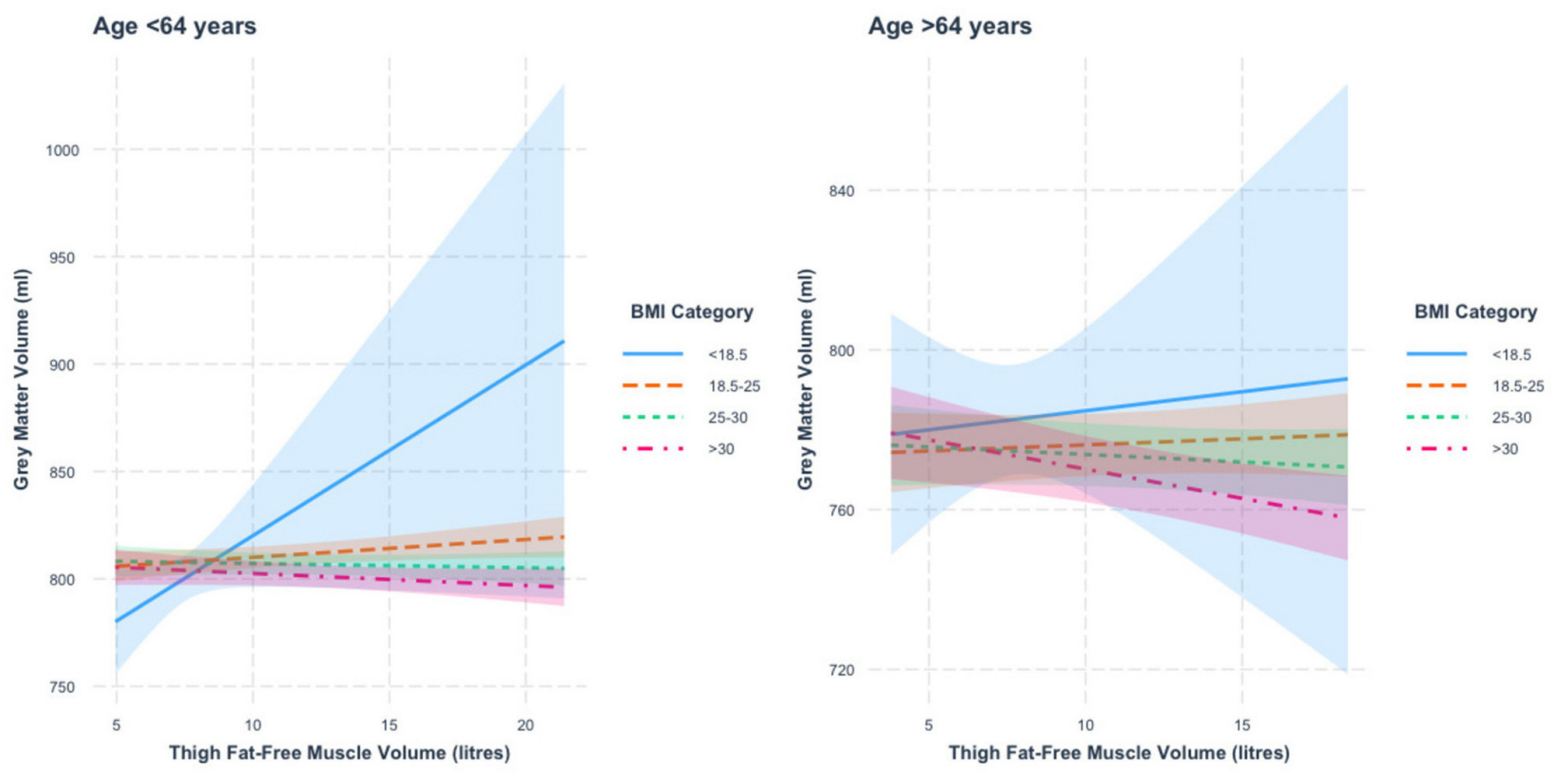
Association between thigh fat-free muscle volume (FFMV) and gray matter volume by body mass index (BMI) stratified by age. Shaded areas represent 95% confidence intervals (CIs).

When stratified by age, we found a thigh FFMV × BMI interaction with GMV in those under 64 years of age (*p* = 3.7 × 10^−5^). Figure 2 presents the nature of this interaction. Specifically, we found that the thigh FFMV-GMV association was significantly different in those with BMI 18.5–24.9 from those with BMI 25–29.9 (*p* = 0.007) and BMI >30 (*p* = 0.002). When the analysis was stratified by BMI, we found a similar pattern to the thigh FFMV–TBV association, in that there was a positive association between thigh FFMV and GMV in those with BMI < 18.5 (β = 7.9 ml/L, *p* = 0.07) and BMI 18.5–24.9 (β = 0.8 ml/L, *p* = 0.04), and a negative association in those with BMI 25–29.9 (β = −0.2 ml/L, *p* = 0.59), and BMI ≥30 (β = 0.6 ml/L, *p* = 0.18).

In those 64 years or older, we also found a thigh FFMV × BMI interaction with GMV (*p* = 8.5 × 10^−6^). Figure 2 presents this interaction. We mainly found that the association between thigh FFMV and GMV was significantly different in those with BMI ≥30 when compared to those with BMI 18.5–24.9 (*p* = 0.001) and BMI 25–29.9 (*p* = 0.04). When the analysis was stratified by BMI, we again found a trend toward positive associations in those with low/normal BMI and negative associations in the overweight/obese groups, though only the group with BMI ≥30 was statistically significant (BMI < 18.5, β = 1.0 ml/L, *p* = 0.78; BMI 18.5–24.9, β = 0.3 ml/L, *p* = 0.51; BMI 25–29.9, β = −0.4 ml/L, *p* = 0.37; BMI ≥30, β = −1.5 ml/L, *p* = 0.006). To assist visualization, rescaled graphs, with those with BMI < 18.5 not plotted, are presented in Supplementary Figure 3.

#### 3.1.3 White matter volume

Greater thigh FFMV was overall associated with greater WMV (model 1, β = 1.9 ml/L, *p* < 2 × 10^−16^), although this lost statistical significance when the age × sex interaction was included in the model (model 2, β = 0.04 ml/L, *p* = 0.83). We additionally found an interaction between thigh FFMV and BMI on WMV (model 3, β = −0.07, *p* = 0.003), which persisted when potential confounders were included (model 4, β = −0.08, *p* = 0.004).

To explore the nature of the thigh muscle volume × BMI interaction, we first examined its presence when stratified by sex in fully adjusted models (Supplementary Table 5). There was no thigh FFMV × BMI interaction in women (*p* = 0.89) or in men (*p* = 0.87).

**Figure 3:**
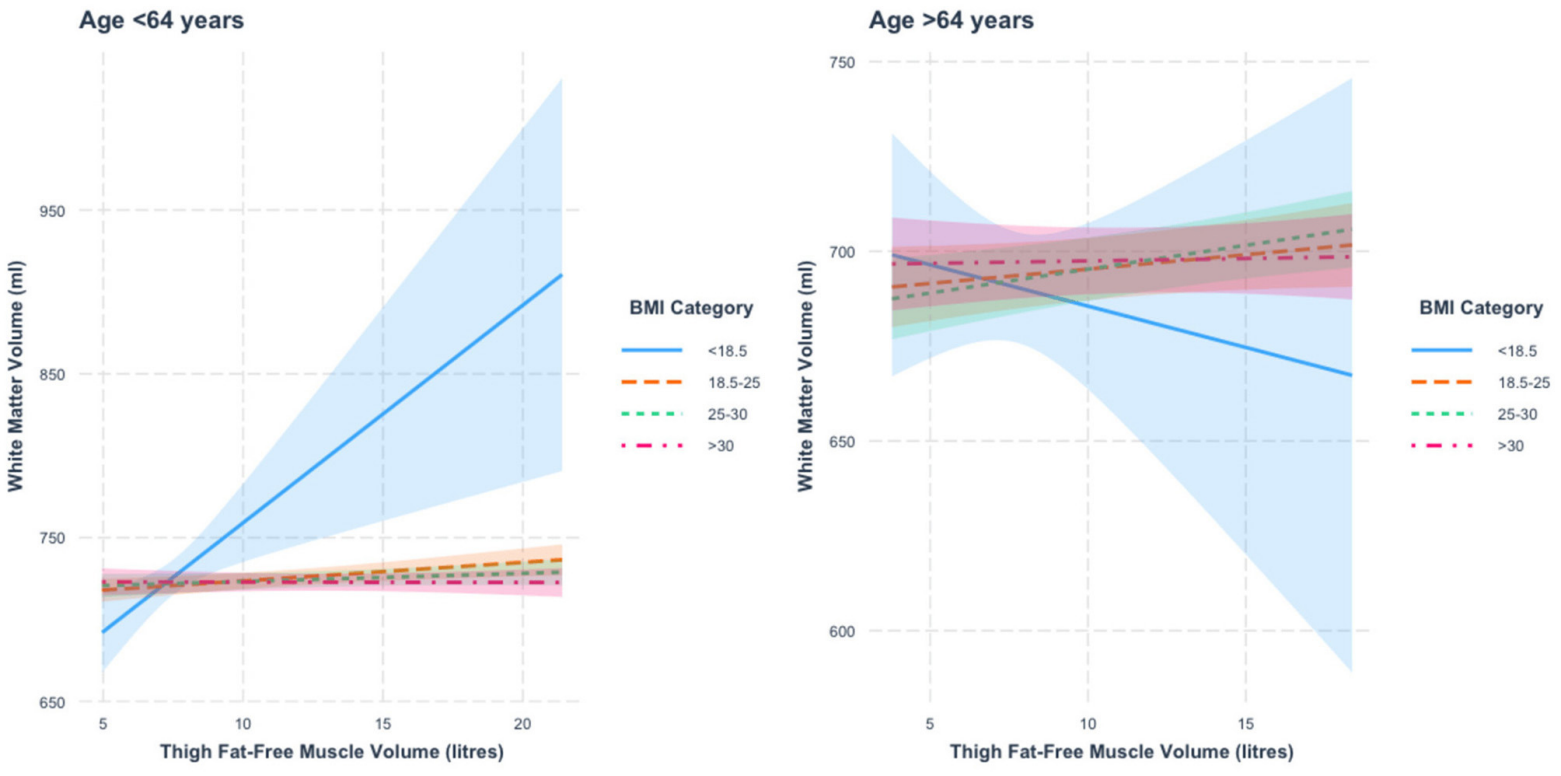
Association between thigh fat-free muscle volume (FFMV) and white matter volume by body mass index (BMI) stratified by age. Shaded areas represent 95% confidence intervals (CIs).

When stratified by age, we found a thigh FFMV × BMI interaction with WMV in those under 64 years of age (*p* = 0.006). Figure 3 presents the nature of this interaction. We found that relative to those with BMI < 18.5, the association between thigh FFMV and WMV was statistically different in the other three BMI strata (all *p* < 0.05). Relative to those with BMI 18.5–24.9, the association was only significantly different in those with BMI < 18.5 and BMI ≥30 (both *p* < 0.05). When the analysis was stratified by BMI, we found a positive thigh FFMV-WMV association that was greatest in those with BMI < 18.5 (β = 13.3 ml/L, *p* = 0.002), followed by those with BMI 18.5–24.9 (β = 1.1 ml/L, *p* = 0.006). Although not statistically significant, there was a possible positive association in those with BMI 25–29.9 (β = 0.5 ml/L, *p* = 0.17) and a negative association in those with BMI ≥30 (β = −0.020 ml/L, *p* = 0.96).

In those 64 years or older, we did not find a thigh FFMV × BMI interaction with WMV (β = −36.4, *p* = 0.44). To assist visualization, rescaled graphs, with those with BMI < 18.5 not plotted, are presented in Supplementary Figure 4.

#### 3.1.4 Total hippocampal volume

Greater thigh FFMV was associated with smaller THV (model 1, β = −0.10 ml/L, *p* < 2 × 10^−16^). The association between thigh FFMV and THV remained when the age × sex interaction was included in the model (model 2, β = −0.06 ml/L, *p* < 2 × 10^−16^). Although we did not find an interaction between thigh FFMV and BMI on THV initially (model 3, β = −0.001, *p* = 0.06), the interaction became statistically significant when potential confounders were included (model 4, β = −0.002, *p* = 0.02).

To explore the nature of the thigh FFMV × BMI interaction, we first examined for the presence of the interaction when stratified by sex in fully adjusted models (Supplementary Table 5). We did not find a thigh FFMV × BMI interaction in women (*p* = 0.11) or men (*p* = 0.29).

When stratified by age, we did not find a thigh FFMV × BMI interaction with THV in those aged below 64 years (*p* = 0.11) or those aged 64 years and older (*p* = 0.20). Supplementary Figure 5 presents the nature of the relationship between thigh FFMV and THV when stratified by age and BMI, and Supplementary Figure 6 presents the associations with those with BMI < 18.5 that are not plotted.

#### 3.1.5 White matter hyperintensity volume

Greater thigh FFMV was associated with smaller WMHV (model 1, β = −0.07 ml/L, *p* = 0.0006). The association between thigh FFMV and WMHV remained when the age × sex interaction was included in the model (model 2, β = 0.09 ml/L, *p* = 0.01). We found an interaction between thigh FFMV and BMI on WMHV (model 3, β = 0.03, *p* = 1.0 × 10^−11^), which persisted when potential confounders were included (model 4, β = 0.03, *p* = 1.3 × 10^−7^).

To explore the nature of the thigh FFMV × BMI interaction, we first examined for the presence of the interaction when stratified by sex in fully adjusted models (Supplementary Table 5). The thigh FFMV × BMI interaction term was statistically significant in women (*p* = 0.02, see Supplementary Figure 7) but not in men (*p* = 0.78). In women, the association between FFMV and WMHV in those with a BMI ≥30 differed from those with a BMI of 18.5–24.9 (*p* = 0.04). The nature of this difference was that there was a positive association between greater FFMV and greater WMHV (β = 0.3 ml/L, *p* = 0.03) in those with BMI ≥30 that was not seen in those with BMI < 20, 20–25, and 25–30 (all *p* > 0.05).

**Figure 4:**
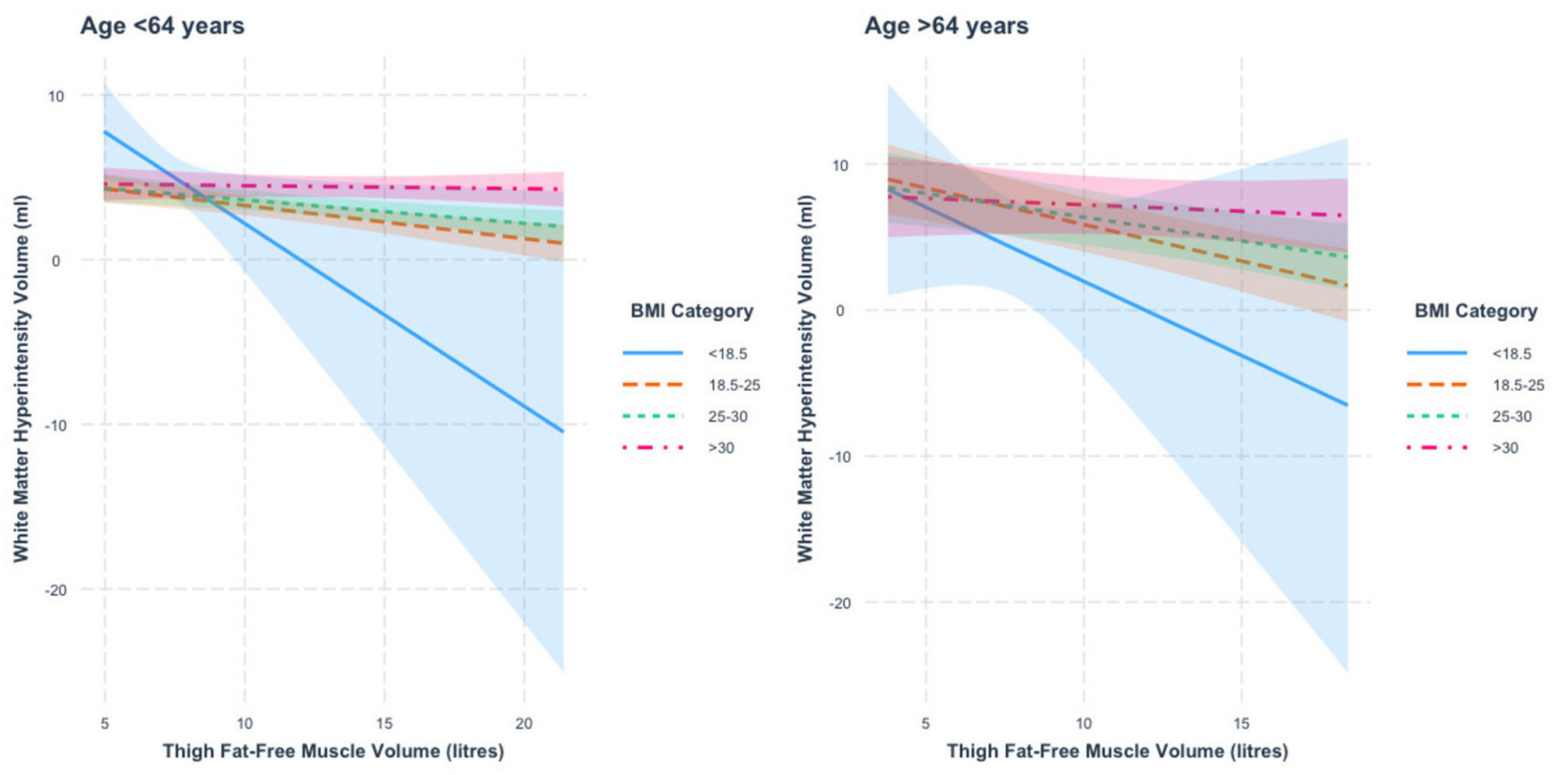
Association between thigh fat-free muscle volume (FFMV) and white matter hyperintensity volume by BMI stratified by age. Shaded areas represent 95% confidence intervals (CIs).

When stratified by age, we found a thigh FFMV × BMI interaction in those < 64 years of age (*p* = 1.2 × 10^−6^). Figure 4 presents the nature of this interaction. We found that the association between thigh FFMV and WMHV was significantly different in those with BMI ≥30 relative to those with BMI < 18.5 (*p* = 0.04), BMI 18.5–24.9 (*p* = 0.0008) and BMI 25–29.9 (*p* = 0.02). When stratified by BMI, we found negative thigh FFMV–WMHV associations that were largest in those with BMI < 18.5 (β = −1.1 ml/L, *p* = 0.04), followed by those with BMI 18.5–24.9 (β = −0.2 ml/L, *p* = 3.7 × 10^−5^) and BMI 25–29.9 (β = −0.1 ml/L, *p* = 0.001). There was also a negative association in those with BMI ≥30 (β = −0.02 ml/L, *p* = 0.71) that was not statistically significant.

In those 64 years or older, we also found a thigh FFMV × BMI interaction with WMHV (*p* = 0.0009). Figure 4 presents this interaction. We found that the association between thigh FFMV and WMHV was significantly different in those with BMI ≥30 relative to those with BMI 18.5–24.9 (*p* = 0.002). When stratified by BMI, we found negative associations between thigh FFMV and WMHV in all BMI strata, although they were only significant in those with BMI 18.5–24.9 (β = −0.5 ml/L, *p* = 6.9 × 10^−6^) and BMI 25–29.9 (β = −0.3 ml/L, *p* = 0.001). To assist visualization, rescaled graphs, with those with BMI < 18.5 not plotted, are presented in Supplementary Figure 8.

### 3.2 Apolipoprotein E e4 status and brain volumes

The results of repeating the above analyses in those carrying an ApoE e4 allele (*n* = 5,485, 26.9% of the whole cohort) are presented in Supplementary Table 6. In this group, greater thigh FFMV was associated with smaller TBV (β = −1.9 ml/L, *p* < 0.05), smaller GMV (β = −4.0 ml/L, *p* < 0.05), greater WMV (β = 2.0 ml/L, *p* < 0.05), smaller THV (β = −0.1 ml/L, *p* < 0.05), and smaller WMHV (β = −0.04 ml/L, *p* < 0.05). Similar to the whole cohort, we found thigh FFMV × BMI interactions for TBV and GMV (*p* < 0.05). Initially, an FFMV–BMI interaction for WMHV was no longer statistically significant in the fully adjusted model. Conversely, a FFMV × BMI interaction was found for THV only in the fully adjusted model.

Repeating the above analyses in those not carrying an ApoE e4 allele (73% of the whole cohort), we found similar patterns of associations as those reported in the whole group (Supplementary Table 7).

## 4 Discussion

In this large population-based sample, we found that the relationship between thigh FFMV and brain structure was dependent on BMI and age, but not sex. In those with low or normal BMI, thigh FFMV was positively associated with neuroimaging indices of brain health. However, this association diminished with greater BMI, becoming negative in those who were overweight or obese. This broad pattern was more pronounced in the younger group and was independent of cardiometabolic risk factors and physical activity. Our results suggest that greater muscle volume may be beneficial for brain health, but this benefit may be affected by factors related to obesity and greater age.

The link between sarcopenia and brain health is well-studied, with sarcopenia associated with poorer cognition in people without dementia (Szlejf et al., 2019; Tolea and Galvin, 2015), increased dementia risk (Beeri et al., 2021; Li, 2023), and poorer brain volumes (Burns et al., 2010; Yu et al., 2021). Until recently, it was unclear whether skeletal muscle volume is independently associated with brain health in individuals without sarcopenia. Recent large-scale studies have demonstrated that lower muscle volume is independently associated with increased gray matter atrophy (Yu et al., 2021), greater cognitive decline (Tessier et al., 2022), and greater risk of Alzheimer's dementia (Daghlas et al., 2023). These findings suggest that the interplay between lean muscle and cognition exists outside of sarcopenia. Mechanisms for this remain unclear, but skeletal muscle is increasingly recognized for its role in cognitive function, possibly through myokines and myometabolites that reduce neuroinflammation, improve brain glucose metabolism, and enhance tissue oxidative capacity (Rai and Demontis, 2022; Severinsen and Pedersen, 2020). Reduced or abnormal myokine secretion in those with low muscle volume (Han et al., 2023; Jo et al., 2022) may explain the associations seen with poorer brain health. Our findings suggest that greater muscle volume is associated with better brain structure in those without sarcopenia, particularly those with low or normal BMI, suggesting that increasing muscle volume in mid-to-later life may offer cognitive benefits beyond avoiding sarcopenia.

We found that associations between muscle volume and brain volume differed by BMI and age. Greater thigh FFMV was associated with better TBV, GMV, WMV, and WMHV in those with BMI < 25, particularly in those under 64 years of age. To the best of our knowledge, this has not been previously published. Although the magnitude of these associations was greatest in the group with BMI < 18.5, which makes up only 0.7% of our study population, the presence of similar, but less pronounced findings in those with normal BMI 18.5–24.9 is reassuring. Previous studies examining the relationship between muscle and brain health have not explored whether associations differ by BMI and age (Kilgour et al., 2013; Burns et al., 2010; Gurholt et al., 2021; Weise et al., 2013; Yu et al., 2021). However, this is an important consideration since the mechanisms through which muscle volume, BMI, and age may contribute to brain health may overlap substantially. The muscle-brain relationship may depend on the balance between the hypothesized benefits of greater muscle volume, and the pro-inflammatory and pro-oxidative stress states associated with greater BMI (Uranga and Keller, 2019) and age (Franceschi et al., 2018). Although this hypothesis requires testing, our results suggest that certain groups may benefit from muscle-building interventions. In contrast, others may benefit from BMI-reduction interventions, recognizing that these are not mutually exclusive.

Our finding that greater FFMV was associated with poorer TBV and GMV in those with BMI > 30 was unexpected. A possible explanation may be related to the measurement method of FFMV used in the UK Biobank. Voxels with a fat fraction of < 50% are defined as fat-free muscle in this cohort. It is possible that small pockets/streaks of fatty infiltration of muscle are included in calculations of total muscle volume, resulting in intramuscular fat contributing to an overestimation of lean muscle volume. This may explain why we were more likely to observe a null or negative association between FFMV and brain volumes in the obese groups or those older than 64 years, given intramuscular fat increases with obesity (Hilton et al., 2008) and age (Dai et al., 2023; Figueiredo et al., 2021; Hogrel et al., 2015). Reverse causation is another plausible explanation. Obesity is associated with poorer cognitive outcomes (Singh-Manoux et al., 2012), and cognitive dysfunction has also been linked to overeating and obesity (Gunstad et al., 2020). It is possible that individuals with obesity might have smaller brain volumes alongside greater muscle volume due to the negative effects of adiposity or preceding cognitive impairment, especially if the FFMV measure captures intramuscular fat. This cannot be ruled out on cross-sectional analysis, and longitudinal analysis is required to determine the direction of causality.

Unexpectedly, we observed a negative association between thigh FFMV and THV across all BMI categories in patients under 64 years of age. One possible explanation is that our analysis did not account for potential unrecognized confounders. Although exploring the underlying mechanisms behind this observed relationship was beyond this study's scope, it further highlights the complexity of the interplay between muscle volume and brain structure, emphasizing the need for future studies to unpack these nuances.

The key strengths of this study include the large sample size with well-characterized brain and abdominal MRI data, providing excellent statistical power to explore interactions with BMI, sex, and age. However, the study has several limitations. Being volunteer based, the UK Biobank cohort may not accurately represent the general population, with overall higher education and socioeconomic status, lower overall rates of cardiometabolic conditions, and relative racial homogeneity. The cross-sectional nature of the analysis prevents causal inference, and reverse causation cannot be ruled out. Additionally, BMI as a proxy for adiposity has limitations, as it does not differentiate between fat and muscle volume. Future study is needed to determine if the signals we report exist longitudinally or are adjustable to improve or maintain brain health.

## 5 Conclusion

This large-scale cross-sectional study found that greater thigh FFMV is linked to better volumetric indices of brain health at midlife in individuals without sarcopenia. However, this association appears to diminish with increasing BMI and age. Further research is required to establish causal relationships between muscle volume and brain health, as well as the mechanisms underlying any potential links.

## Data Availability

The data analyzed in this study is subject to the following licenses/restrictions: the data analyzed in this study are available from the UK Biobank upon reasonable request. Requests to access these datasets should be directed to https://www.ukbiobank.ac.uk/enable-your-research/apply-for-access.
