# Supplementary material for: Interactions between muscle volume and body mass index on brain structure in the UK Biobank
Source: Front Dement. 2024 Sep 23;3:1456716. doi: 10.3389/frdem.2024.1456716 (PMC11456486; doi:10.3389/frdem.2024.1456716)
Supplement: Supplementary file 1 [file Table_1.DOCX]

Supplementary Material

Supplementary Table 1 Characteristics of participants with and without possible sarcopenia

|  | **Whole group**  **Mean +/- SD *or* median (IQR) *or* n (%)** | **Without possible Sarcopenia** | **With possible sarcopenia** |
| --- | --- | --- | --- |
| n | 21660 | 20353 | 1307 |
| Age (years) | 64 (57 to 69) | 64 (57 to 69) | 68 (62 to 72) |
| Female sex | 11547 (53.3) | 10808 (53.1) | 739 (56.5) |
| Townsend Deprivation Index | -2.7 (-4.0 to -0.7) | -2.7 (-4.0 to -0.8) | -2.6 (-3.8 to -0.3) |
| Tertiary education | 10116 (46.7) | 9606 (47.2) | 510 (39.0) |
| White ethnicity | 21005 (97.0) | 19749 (97.0) | 1256 (96.1) |
| Ever smoked | 11354 (52.4) | 10672 (52.4) | 682 (52.2) |
| Current or previous alcohol drinker | 20821 (96.1) | 19584 (96.2) | 1237 (94.6) |
| Sleep duration (hours) | 7 (7 to 8) | 7 (7 to 8) | 7 (6 to 8) |
| Depressed mood in last 2 weeks | 3718 (17.2) | 3146 (15.5) | 248 (19.0) |
| Hypertension | 8112 (37.5) | 7487 (36.8) | 625 (47.8) |
| Diabetes | 1159 (5.4) | 1028 (5.1) | 131 (10.0) |
| Hypercholesterolemia | 5104 (23.6) | 4657 (22.9) | 447 (34.2) |
| Ischemic Heart Disease | 1162 (5.4) | 1042 (5.1) | 120 9.2) |
| Stroke | 797 (3.7) | 725 (3.6) | 72 (5.5) |
| BMI (kg/m²)  <18.5  18.5 to 24.9  25 to 29.9  ≥30 | 26.5 ± 4.3  154 (0.7)  8632 (39.9)  8916 (41.2)  3878 (17.9) | 26.5 ± 4.3  138 (0.7)  8150 (40.0)  8398 (41.3)  3595 (17.7) | 26.9 ± 4.7  16 (1.2)  482 (36.9)  518 (39.6)  283 (21.7) |
| Systolic blood pressure (mmHg) | 139.1 ± 19.2 | 139.0 ± 19.2 | 140.0 ± 19.2 |
| Diastolic blood pressure (mmHg) | 78.5 ± 10.6 | 78.6 ± 10.6 | 77.1 ± 10.5 |
| Physical activity |  |  |  |
| Low | 3446 (15.9) | 3202 (15.7) | 244 (18.7) |
| Moderate | 7614 (35.2) | 7148 (35.1) | 466 (35.7) |
| High | 7227 (33.4) | 6862 (33.7) | 365 (27.9) |
| Total MET minutes per week | 1704 (809 to 3276) | 1716 (815 to 3279) | 1525 (660 to 3066) |
| Walking pace |  |  |  |
| Slow (<3 miles/hour) | 886 (4.1) | 722 (3.6) | 164 (12.5) |
| Steady (3-4 miles/hour) | 10698 (49.4) | 9982 (49.0) | 716 (54.8) |
| Brisk (>4 miles/hour) | 9926 (45.8) | 9520 (46.8) | 406 (31.1) |
| Right hand grip strength (kg) | 30 (24 to 39) | 30.0 (24 to 40) | 15 (12 to 22) |
| Apolipoprotein E ɛ4 carrier | 5809 (26.8) | 5485 (26.9) | 324 (24.8) |
| Total thigh fat-free muscle volume (L) | 9.7 (8.1 to 12.2) | 9.8 (8.1 to 12.2) | 8.9 (7.4 to 11.0) |
| Total brain volume (ml) | 1501.3 +/- 73.0 | 1502.7 ± 72.6 | 1477.7 ± 74.3 |
| Grey matter volume (ml) | 795.5 +/- 48.1 | 796.4 ± 47.8 | 781.0 ± 50.7 |
| White matter volume (ml) | 705.8 +/- 41.0 | 706.3 ± 40.8 | 698.0 ± 42.2 |
| Total hippocampal volume (ml) | 10.0 +/- 1.1 | 10.0 ± 1.1 | 9.8 ± 1.2 |
| Total volume of white matter hyperintensities (ml) | 3.5 (1.9 to 7.0) | 3.4 (1.9 to 6.8) | 4.8 (2.6 to 9.3) |

***Key****: n, number; IQR, interquartile range; SD, standard deviation; BMI, body mass index; MET, metabolic equivalent of task*

Supplementary Table 2 Characteristics of non-sarcopenic participants with and without ApoE e4 allele

|  | **Non-sarcopenic group**  **Mean +/- SD *or* median (IQR) *or* n (%)** | **ApoE ɛ4 positive** | **ApoE ɛ4 negative** |
| --- | --- | --- | --- |
| n | 20353 | 5485 | 14319 |
| Age (years) | 64 (57 to 69) | 63.2 (57 to 69) | 63.8 (57 to 69) |
| Female sex | 10808 (53.1%) | 2972 (54.2%) | 7506 (52.4%) |
| Townsend Deprivation Index | -2.7 (-4.0 to -0.8) | -2.7 (-4.0 to -0.8) | -2.7 (-4.0 to -0.8) |
| Tertiary education | 9606 (47.2%) | 2621 (47.8%) | 6718 (46.9%) |
| White ethnicity | 19749 (97.0%) | 5330 (97.2%) | 13894 (97.0%) |
| Ever smoked | 10672 (52.4%) | 2871(52.3%) | 7515 (52.5%) |
| Current or previous alcohol drinker | 19584 (96.2%) | 5264 (96.0%) | 13793 (96.3%) |
| Sleep duration (hours) | 7 (7 to 8) | 7 (6 to 8) | 7 (7 to 8) |
| Depressed mood in last 2 weeks | 3146 (15.5%) | 994 (18.1%) | 2389 (16.7%) |
| Hypertension | 7487 (36.8%) | 1986 (36.2%) | 5305 (37.0%) |
| Diabetes | 1028 (5.1%) | 265 (4.8%) | 734 (5.1%) |
| Hypercholesterolemia | 4657 (22.9%) | 1392 (25.4%) | 3132 (21.9%) |
| Ischemic Heart Disease | 1042 (5.1%) | 307 (5.6%) | 713 (5.0%) |
| Stroke | 725 (3.6%) | 209 (3.8%) | 501 (3.5%) |
| BMI (kg/m²)  <18.5  18.5 to 24.9  25 to 29.9  ≥30 | 26.5 ± 4.3  138 (0.7%)  8150 (40.0%)  8398 (41.3%)  3595 (17.7%) | 26.4 ± 4.3  45 (0.8%)  2235 (40.7%)  2189 (39.9%)  996 (18.2%) | 26.5 ± 4.3  89 (0.6%)  5691 (39.7%)  5989 (41.8%)  2500 (17.5%) |
| Systolic blood pressure (mmHg) | 139.0 ± 19.2 | 138.6 ± 19.5 | 139.1 ± 19.0 |
| Diastolic blood pressure (mmHg) | 78.6 ± 10.6 | 78.5 ± 10.7 | 78.7 ± 10.5 |
| Physical activity |  |  |  |
| Low | 3202 (15.7%) | 861 (16.7%) | 2294 (16.0%) |
| Moderate | 7148 (35.1%) | 1947 (35.5%) | 5075 (35.4%) |
| High | 6862 (33.7%) | 1869 (34.1%) | 4865 (34.0%) |
| Total MET minutes per week | 1716 (815 to 3279) | 1710 (810 to 3306) | 1710 (815 to 3270) |
| Walking pace |  |  |  |
| Slow (<3 miles/hour) | 722 (3.6%) | 175 (3.2%) | 528 (3.7%) |
| Steady (3-4 miles/hour) | 9982 (49.0%) | 2665 (48.6%) | 7051 (49.2%) |
| Brisk (>4 miles/hour) | 9520 (46.8%) | 2605 (47.5%) | 6655 (46.5%) |
| Right hand grip strength (kg) | 30.0 (24 to 40) | 30.0 (24 to 40) | 30 (24 to 40) |
| Apolipoprotein E e4 carrier | 5485 (26.9%) | NA | NA |
| Total thigh fat-free muscle volume (L) | 9.8 (8.1 to 12.2) | 9.7 (8.1 to 12.2) | 9.9 (8.1 to 12.3) |
| Total brain volume (ml) | 1502.7 ± 72.6 | 1505.0 ± 72.5 | 1501.9 ± 72.5 |
| Grey matter volume (ml) | 796.4 ± 47.8 | 798.3 ± 47.5 | 795.7 ± 47.9 |
| White matter volume (ml) | 706.3 ± 40.8 | 706.7 ± 40.5 | 706.2 ± 40.9 |
| Total hippocampal volume (ml) | 10.0 ± 1.1 | 9.9 ± 1.1 | 10.1 ± 1.1 |
| Total volume of white matter hyperintensities (ml) | 3.4 (1.9 to 6.8) | 3.4 (1.9 to 6.8) | 3.5 (1.8 to 6.8) |

***Key****: ApoE, apolipoprotein E; n, number; IQR, interquartile range; SD, standard deviation; BMI, body mass index; MET, metabolic equivalent of task*

Supplementary Table 3 Comparative analysis of thigh fat-free muscle volume associations with brain volumes across body mass index categories

| **Brain Region** | **Age Category** | **BMI Reference group** | **β-coefficient for BMI group against reference group (p-value)** | | | |
| --- | --- | --- | --- | --- | --- | --- |
|  |  |  | **<18.5** | **18.5 – 24.9** | **25 – 29.9** | **≥30** |
| **Total brain volume** | <64yrs | <18.5 | N/A | -19.3  (p=0.008*) | -20.9  (p=0.004*) | -21.8  (p=0.003*) |
|  |  | 18.5-24.9 | 19.3  (p=0.008*) | N/A | -1.7  (p=0.009*) | -2.5  (p=0.0007*) |
|  |  | 25-29.9 | 20.9  (p=0.004*) | 1.7  (p=0.009*) | N/A | -0.9  (p=0.21) |
|  | ≥64yrs | <18.5 | N/A | 2.3  (p=0.69) | 2.1  (p=0.71) | -0.1  (p=0.98) |
|  |  | 18.5-24.9 | -2.3  (p=0.69) | N/A | -0.2  (p=0.80) | -2.4  (p=0.008*) |
|  |  | 25-29.9 | -2.1  (p=0.71) | 0.2  (p=0.80) | N/A | -2.2  (p=0.01*) |
| **Grey matter volume** | <64yrs | <18.5 | N/A | -7.1  (p=0.10) | -8.1  (p=0.06) | -8.5  (p=0.05) |
|  |  | 18.5-24.9 | 7.1  (p=0.10) | N/A | -1.0  (p=0.007*) | -1.4  (p=0.002*) |
|  |  | 25-29.9 | 8.1  (p=0.06) | 1.0  (p=0.007*) | N/A | -0.4  (p=0.39) |
|  | ≥64yrs | <18.5 | N/A | -0.7  (p=0.85) | -1.3  (p=0.70) | -2.4  (p=0.49) |
|  |  | 18.5-24.9 | 0.7  (p=0.85) | N/A | -0.7  (p=0.12) | -1.8  (p=0.001*) |
|  |  | 25-29.9 | 1.3  (p=0.70) | 0.7  (p=0.12) | N/A | -1.1  (p=0.04*) |
| **White matter volume** | <64yrs | <18.5 | N/A | -12.2  (p=0.006*) | -12.8  (p=0.004*) | -13.3  (p=0.002*) |
|  |  | 18.5-24.9 | 12.2 (p=0.006*) | N/A | -0.6  (p=0.10) | -1.2  (p=0.01*) |
|  |  | 25-29.9 | 12.8  (p=0.004*) | 0.6  (p=0.10) | N/A | -0.5  (p=0.23) |
|  | ≥64yrs | <18.5 | N/A | 2.9  (p=0.43) | 3.4  (p=0.35) | 2.3  (p=0.53) |
|  |  | 18.5-24.9 | -2.9  (p=0.43) | N/A | 0.5  (p=0.28) | -0.6  (p=0.28) |
|  |  | 25-29.9 | -3.4  (p=0.35) | -0.5  (p=0.28) | N/A | -1.1  (p=0.04*) |
| **Total hippocampal volume** | <64yrs | <18.5 | N/A | 0.2  (p=0.08) | 0.2  (p=0.08) | 0.2  (p=0.10) |
|  |  | 18.5-24.9 | -0.2  (p=0.08) | N/A | -0.0004  (p=0.97) | -0.01  (p=0.37) |
|  |  | 25-29.9 | -0.2  (p=0.08) | 0.0004  (p=0.97) | N/A | -0.01  (p=0.35) |
|  | ≥64yrs | <18.5 | N/A | 0.09  (p=0.36) | 0.08  (p=0.39) | 0.07  (p=0.50) |
|  |  | 18.5-24.9 | -0.09  (p=0.36) | N/A | -0.006  (p=0.66) | -0.02  (p=0.13) |
|  |  | 25-29.9 | -0.08  (p=0.39) | 0.006  (p=0.66) | N/A | -0.02  (p=0.23) |
| **White matter hyperintensity volume** | <64yrs | <18.5 | N/A | 0.9  (0.09) | 1.0  (p=0.07) | 1.1  (0.04*) |
|  |  | 18.5-24.9 | -0.9  (p=0.09) | N/A | 0.06  (p=0.20) | 0.2  (p=0.0008*) |
|  |  | 25-29.9 | -1.0  (p=0.07) | -0.06  (p=0.20) | N/A | 0.1  (p=0.02*) |
|  | ≥64yrs | <18.5 | N/A | 0.5  (p=0.55) | 0.7  (p=0.42) | 0.9  (p=0.28) |
|  |  | 18.5-24.9 | -0.5  (p=0.55) | N/A | 0.2  (p=0.09) | 0.4  (p=0.002*) |
|  |  | 25-29.9 | -0.7  (p=0.42) | -0.2  (p=0.09) | N/A | 0.2  (p=0.06) |

***Key****: N/A, not applicable; BMI, body mass index*

** indicates p<0.05*

Supplementary Table 4: Associations between brain volume and thigh fat-free muscle volume stratified by age and BMI

| **Brain Region** | **Stratification by age and BMI in fully adjusted models^a^** | **β-coefficient for association between brain volume and FFMV (ml/L)** | **p-value** |
| --- | --- | --- | --- |
| **Total brain volume** | Age <64 years  BMI <18.5  18.5 – 24.9  25 – 30  ≥30 | 21.2  2.0  0.3  -0.6 | 0.003*  0.004*  0.6  0.40 |
|  | Age ≥64 years  BMI <18.5  18.5 – 24.9  25 – 30  ≥30 | -1.2  1.1  0.9  -1.3 | 0.83  0.17  0.20  0.12 |
| **Grey matter volume** | Age <64 years  BMI <18.5  18.5 – 24.9  25 – 30  ≥30 | 7.9  0.8  -0.2  -0.6 | 0.07  0.04*  0.59  0.18 |
|  | Age ≥64 years  BMI <18.5  18.5 – 24.9  25 – 30  ≥30 | 1.0  0.3  -0.4  -1.5 | 0.78  0.51  0.37  0.006* |
| **White matter volume** | Age <64 years  BMI <18.5  18.5 – 24.9  25 – 30  ≥30 | 13.3  1.1  0.5  -0.02 | 0.002*  0.006*  0.17  0.96 |
|  | Age ≥64 years  BMI <18.5  18.5 – 24.9  25 – 30  ≥30 | -2.2  0.8  1.3  0.1 | 0.56  0.12  0.005*  0.82 |
| **Total hippocampal volume** | Age <64 years  BMI <18.5  18.5 – 24.9  25 – 30  ≥30 | -0.2  -0.04  -0.04  -0.05 | 0.03*  4.6×10^-5^*  3.8×10^-6^*  8.3×10^-7^* |
|  | Age ≥64 years  BMI <18.5  18.5 – 24.9  25 – 30  ≥30 | -0.09  0.005  -0.0006  -0.02 | 0.39  0.71  0.96  0.21 |
| **White matter hyperintensity volume** | Age <64 years  BMI <18.5  18.5 – 24.9  25 – 30  ≥30 | -1.1  -0.2  -0.1  -0.02 | 0.04*  3.7×10^-5^*  0.001*  0.71 |
|  | Age ≥64 years  BMI <18.5  18.5 – 24.9  25 – 30  ≥30 | -1.0  -0.5  -0.3  -0.09 | 0.24  6.9×10^-6^* 0.001*  0.48 |

***Key****: BMI, body mass index; FFMV, fat-free muscle volume*

***^a^****Model includes: Townsend Deprivation Index, education, ethnicity, smoking, alcohol, hypertension, diabetes, hypercholesterolemia, ischemic heart disease, stroke, metabolic equivalent minutes of physical activity per week, Apolipoprotein E ɛ4 carrier status*

**indicates p<0.05*

Supplementary Table 5 Interactions between thigh FFMV and BMI on brain volumes stratified by sex

| **Brain Region** | **Sex** | **β for FFMV × BMI interaction (fully adjusted)^a^** | **p-value** |
| --- | --- | --- | --- |
| **Total brain volume** | Female | -0.1 | 0.2 |
|  | Male | 0.07 | 0.41 |
| **Grey matter volume** | Female | -0.1 | 0.03* |
|  | Male | 0.1 | 0.06 |
| **White matter volume** | Female | -0.009 | 0.89 |
|  | Male | -0.01 | 0.87 |
| **Total hippocampal volume** | Female | -0.003 | 0.11 |
|  | Male | 0.002 | 0.29 |
| **White matter hyperintensity volume** | Female | 0.02 | 0.02* |
|  | Male | 0.003 | 0.78 |

***Key:*** *FFMV, Thigh fat free muscle volume;* *BMI, Body Mass Index*

***^a^****Model includes: Townsend Deprivation Index, education, ethnicity, smoking, alcohol, hypertension, diabetes, hypercholesterolemia, ischemic heart disease, stroke, metabolic equivalent minutes of physical activity per week, Apolipoprotein E ɛ4 carrier status*

**indicates p<0.05*

Supplementary Table 6 Associations and interactions between thigh FFMV, BMI, and normalized brain volumes in those carrying an ApoE e4 allele

|  | **Total brain volume**  β for regression model (95% CI) | **Grey matter volume**  β for regression model (95% CI) | **White matter volume**  β for regression model (95% CI) | **Total hippocampal volume**  β for regression model (95% CI) | **White matter hyperintensity volume**  β for regression model (95% CI) |
| --- | --- | --- | --- | --- | --- |
| **Model 1**  (FFMV) | -1.9 (-2.7, -1.9)* | -4.0 (-4.5, -3.5)* | 2.0 (1.6, 2.4)* | -0.1 (-0.1, -0.08)* | -0.04 (-0.1, 0.04) |
| **Model 2**  (FFMV +  age × sex) | -1.8 (-2.9, -0.6)*  -0.7 (-1.1, -0.2)* | -2.0 (-2.7, -1.3)*  -0.6 (-0.8, -0.3)* | 0.2 (-0.5, 1.0)  -0.09 (-0.4, 0.2) | -0.05 (-0.07, -0.03)*  0.02 (0.01, 0.02)* | 0.06 (-0.08, 0.2)  0.01 (-0.04, 0.07) |
| **Model 3**  (FFMV × BMI +  age × sex) | -0.2 (-0.4, -0.07)*  -0.6 (-1.1, -0.2)* | -0.2 (-0.3, -0.08)*  -0.6 (-0.8, -0.3)* | -0.04 (-0.1, 0.05)  -0.08 (-0.4, 0.2) | -0.002 (-0.005, 0.0004)  0.02 (0.01, 0.02)* | 0.02 (0.004, 0.04)*  0.02 (-0.03, 0.07) |
| **Model 4** (fully adjusted)^a^  (FFMV × BMI +  age × sex) | -0.2 (-0.4, -0.08)*  -0.9 (-1.3, -0.4)* | -0.2 (-0.3, -0.08)*  -0.7 (-1.0, -0.4)* | -0.07 (-0.2, 0.04)  -0.2 (-0.5, 0.1)* | -0.004 (-0.007, -0.0008)*  0.02 (0.007, 0.02)* | 0.02 (-0.003, 0.04)  0.02 (-0.04, 0.07) |

***Key:*** *FFMV, fat free muscle volume;* *BMI, Body Mass Index, ApoE: apolipoprotein E*

***^a^****Model includes: Townsend Deprivation Index, education, ethnicity, smoking, alcohol, hypertension, diabetes, hypercholesterolemia, ischemic heart disease, stroke, metabolic equivalent minutes of physical activity per week*

**indicates p<0.05*

Supplementary Table 7 Associations and interactions between thigh FFMV, BMI, and normalized brain volumes in those not carrying an ApoE e4 allele

|  | **Total brain volume**  β for regression model (95% CI) | **Grey matter volume**  β for regression model (95% CI) | **White matter volume**  β for regression model (95% CI) | **Total hippocampal volume**  β for regression model (95% CI) | **White matter hyperintensity volume**  β for regression model (95% CI) |
| --- | --- | --- | --- | --- | --- |
| **Model 1**  (FFMV) | -2.5 (-2.9, -2.0)* | -4.4 (-4.7, -4.1)* | 1.9 (1.7, 2.2)* | -0.1 (-0.1, -0.09)* | -0.08 (-0.1, -0.03)* |
| **Model 2**  (FFMV +  age × sex) | -3.2 (-3.9, -2.5)*  -0.9 (-1.1, -0.6)* | -3.0 (-3.5, -2.6)*  -0.4 (-0.6, -0.3)* | -0.1 (-0.6, 0.3)  -0.4 (-0.6, -0.3)* | -0.06 (-0.07, -0.05)*  0.02 (0.01, 0.02)* | 0.1 (0.02, 0.2)*  0.04 (0.005, 0.07)* |
| **Model 3**  (FFMV × BMI +  age × sex) | -0.3 (-0.3, -0.2)*  -0.9 (-1.1, -0.6)* | -0.2 (-0.2, -0.1)*  -0.4 (-0.6, -0.3)* | -0.09 (-0.1, -0.03)*  -0.4 (-0.6, -0.2)* | -0.001 (-0.003, 0.0004)  0.02 (0.01, 0.02)* | 0.03 (0.02, 0.04)*  0.04 (0.006, 0.07)* |
| **Model 4** (fully adjusted)^a^  (FFMV × BMI +  age × sex) | -0.2 (-0.3, -0.1)*  -0.8 (-1.1, -0.5)* | -0.2 (-0.2, -0.1)*  -0.5 (-0.6, -0.3)* | -0.08 (-0.1, -0.02)*  -0.4 (-0.6, -0.2)* | -0.001 (-0.003, 0.0007)*  0.02 (0.01, 0.02)* | 0.03 (0.02, 0.04)*  0.04 (0.006, 0.08)* |

***Key:*** *FFMV, fat free muscle volume;* *BMI, Body Mass Index; ApoE, apolipoprotein E*

***^a^****Model includes: Townsend Deprivation Index, education, ethnicity, smoking, alcohol, hypertension, diabetes, hypercholesterolemia, ischemic heart disease, stroke, metabolic equivalent minutes of physical activity per week*

**indicates p<0.5*
