# Supplementary figures and images for: Interactions between muscle volume and body mass index on brain structure in the UK Biobank
Source: Front Dement. 2024 Sep 23;3:1456716. doi: 10.3389/frdem.2024.1456716 (PMC11456486; doi:10.3389/frdem.2024.1456716)

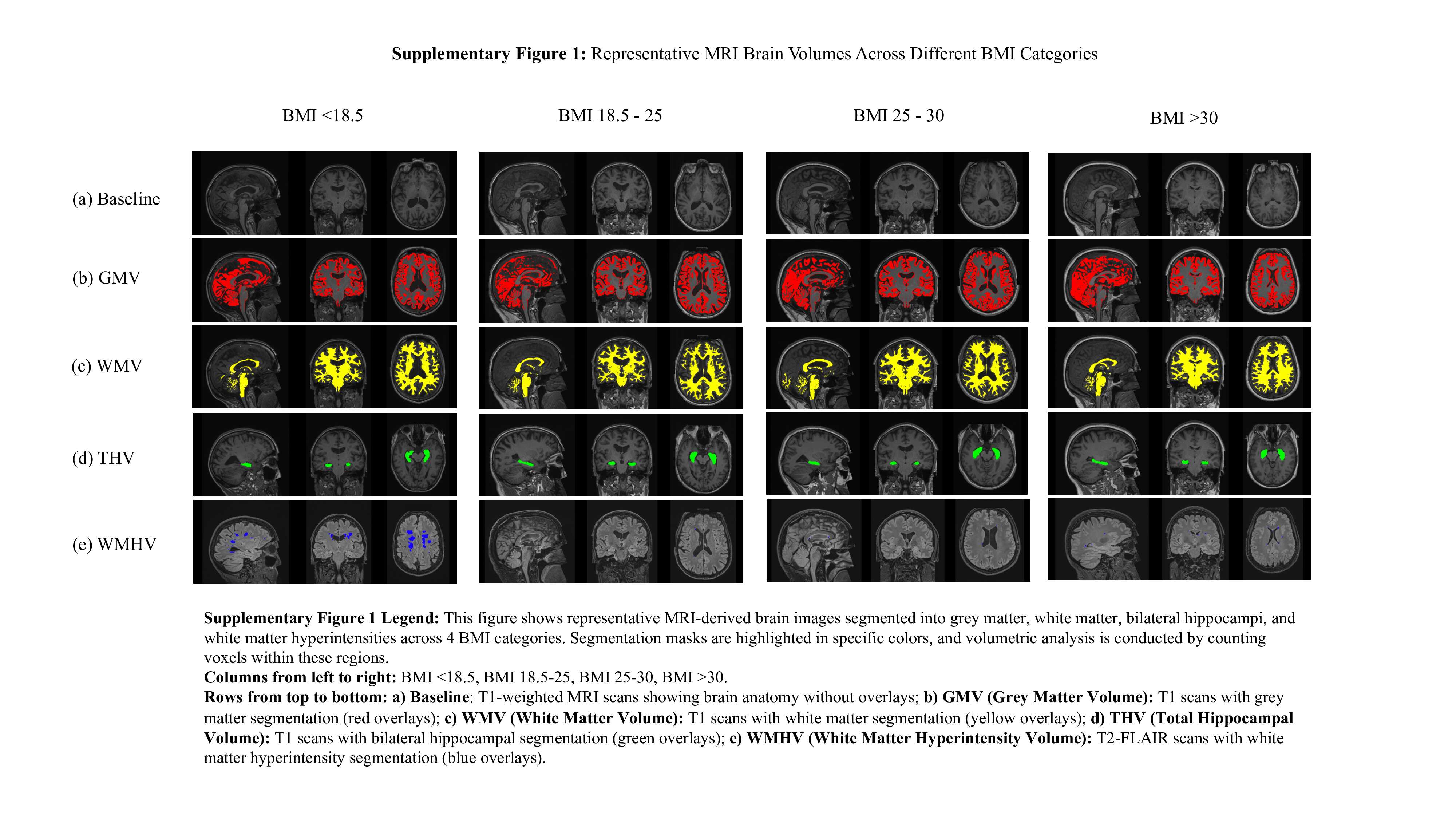

Supplement: Supplementary file 2 [file Image_1.JPEG]

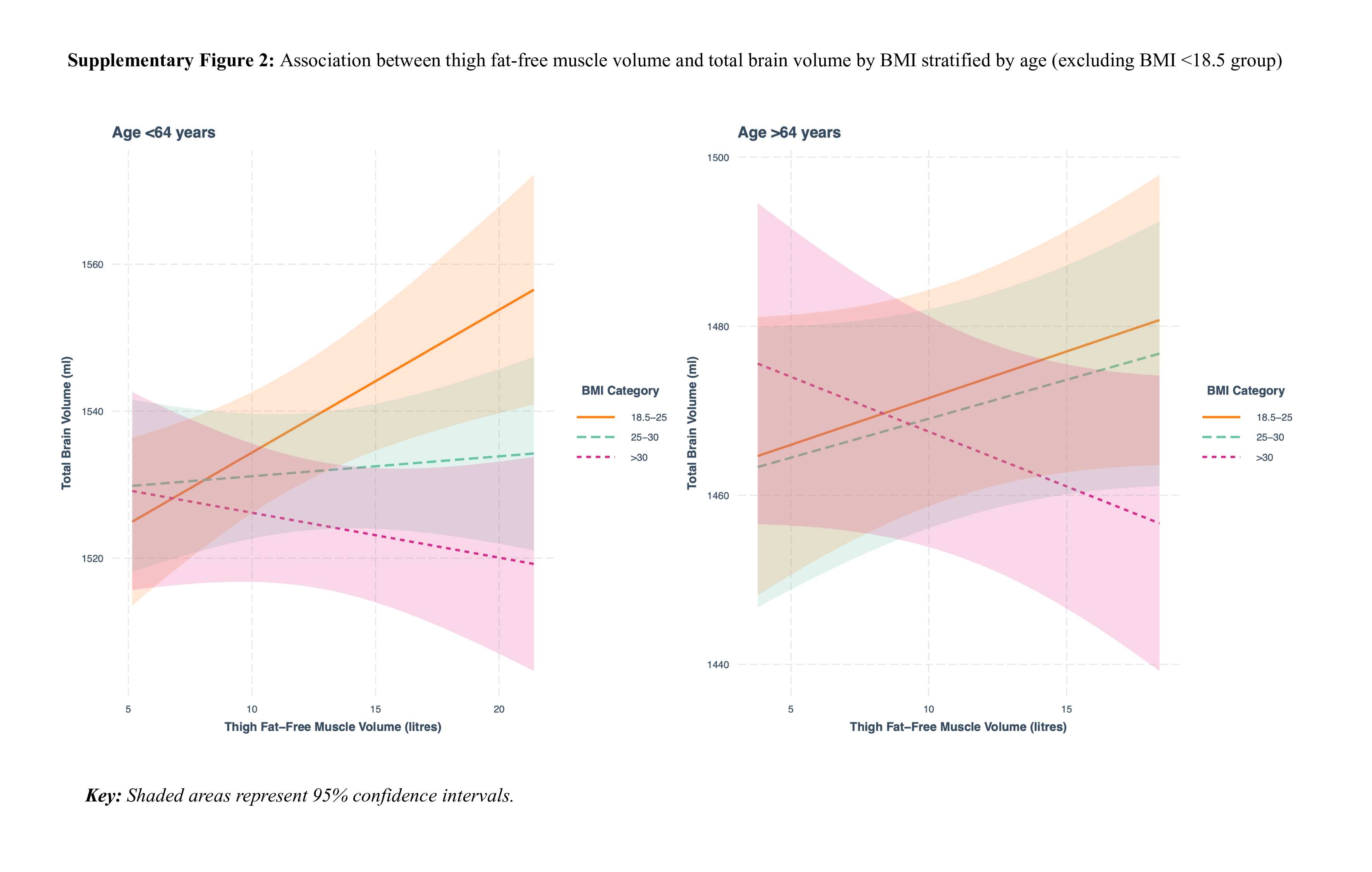

Supplement: Supplementary file 3 [file Image_2.JPEG]

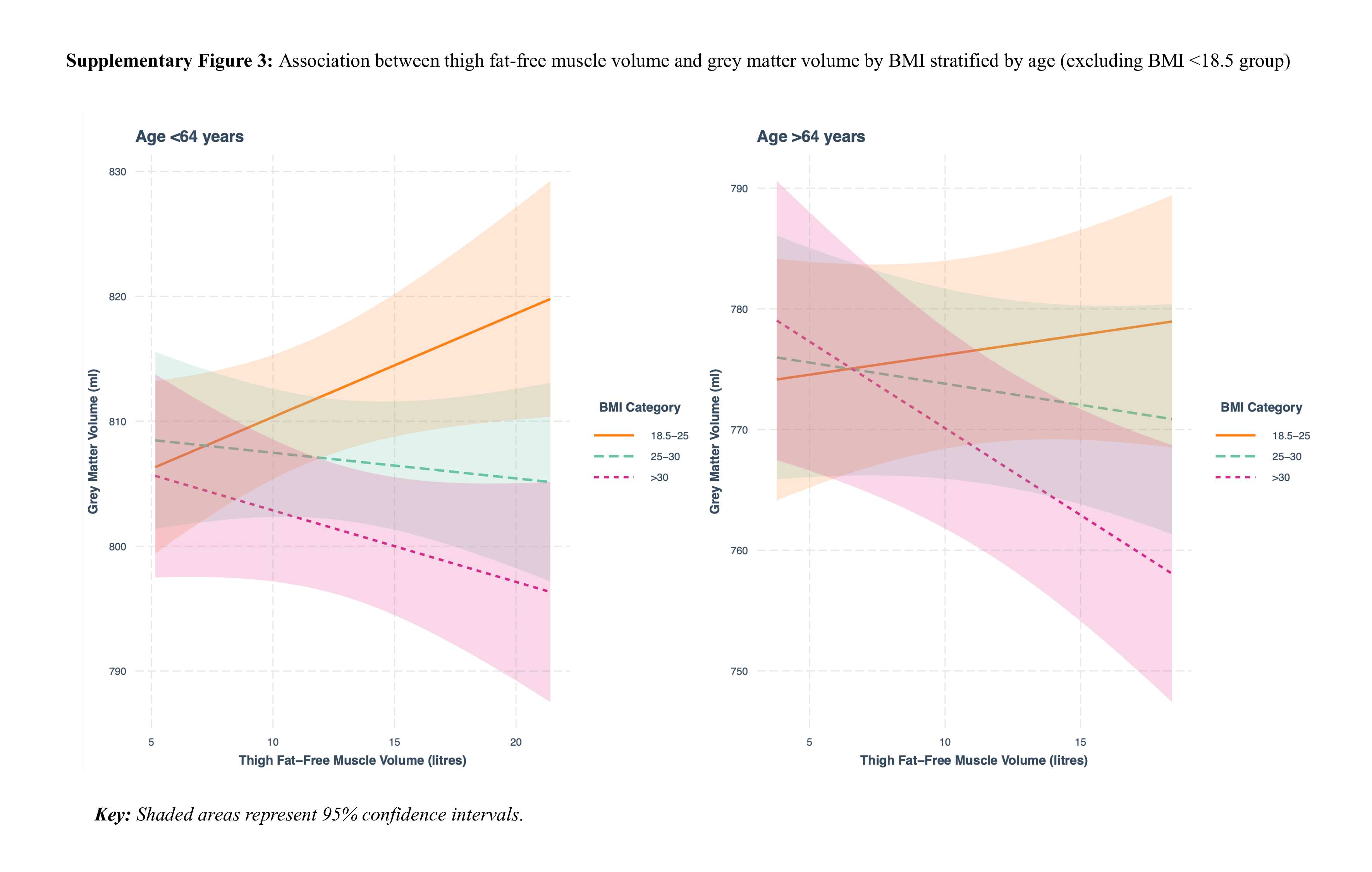

Supplement: Supplementary file 4 [file Image_3.JPEG]

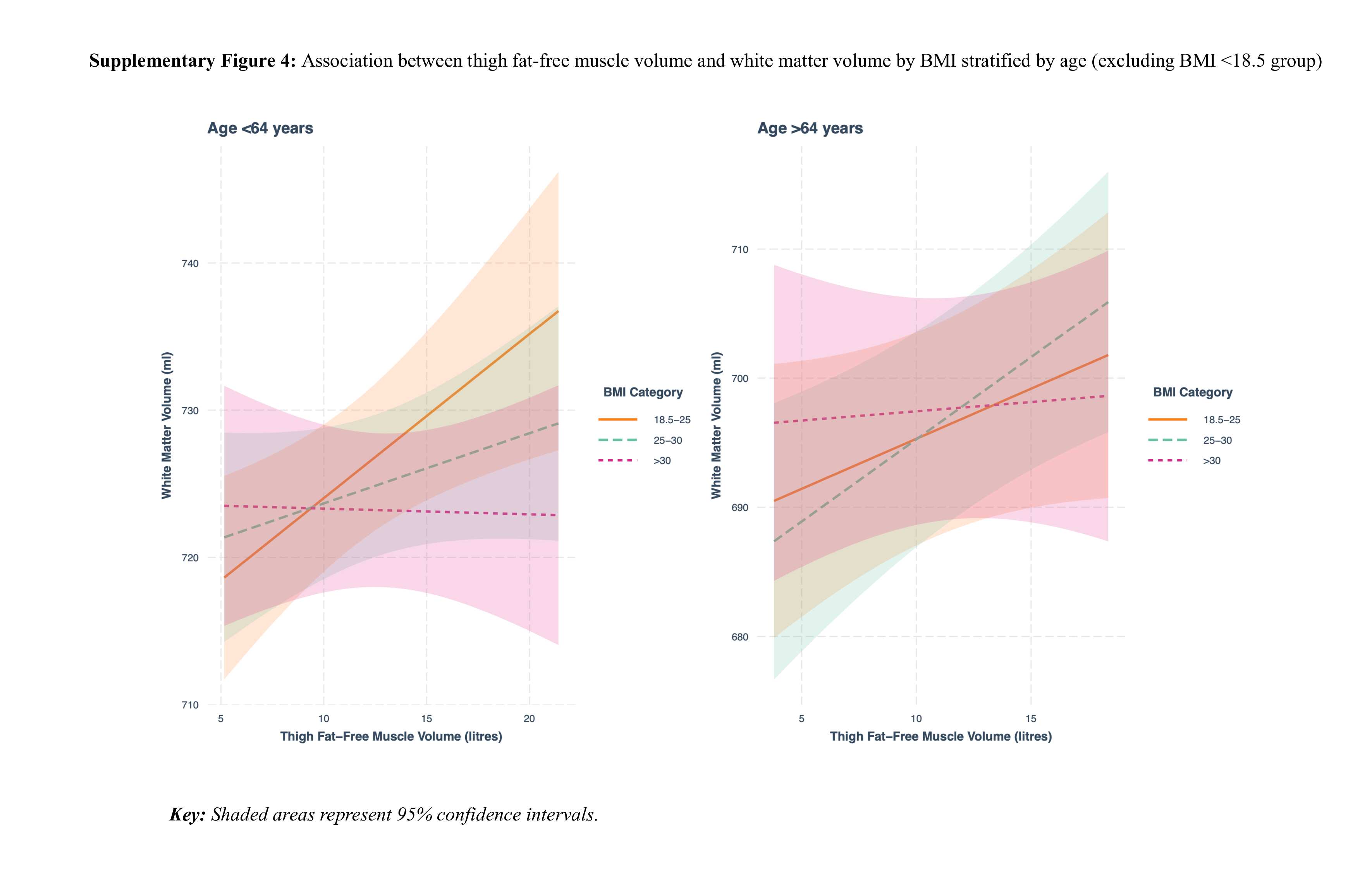

Supplement: Supplementary file 5 [file Image_4.JPEG]

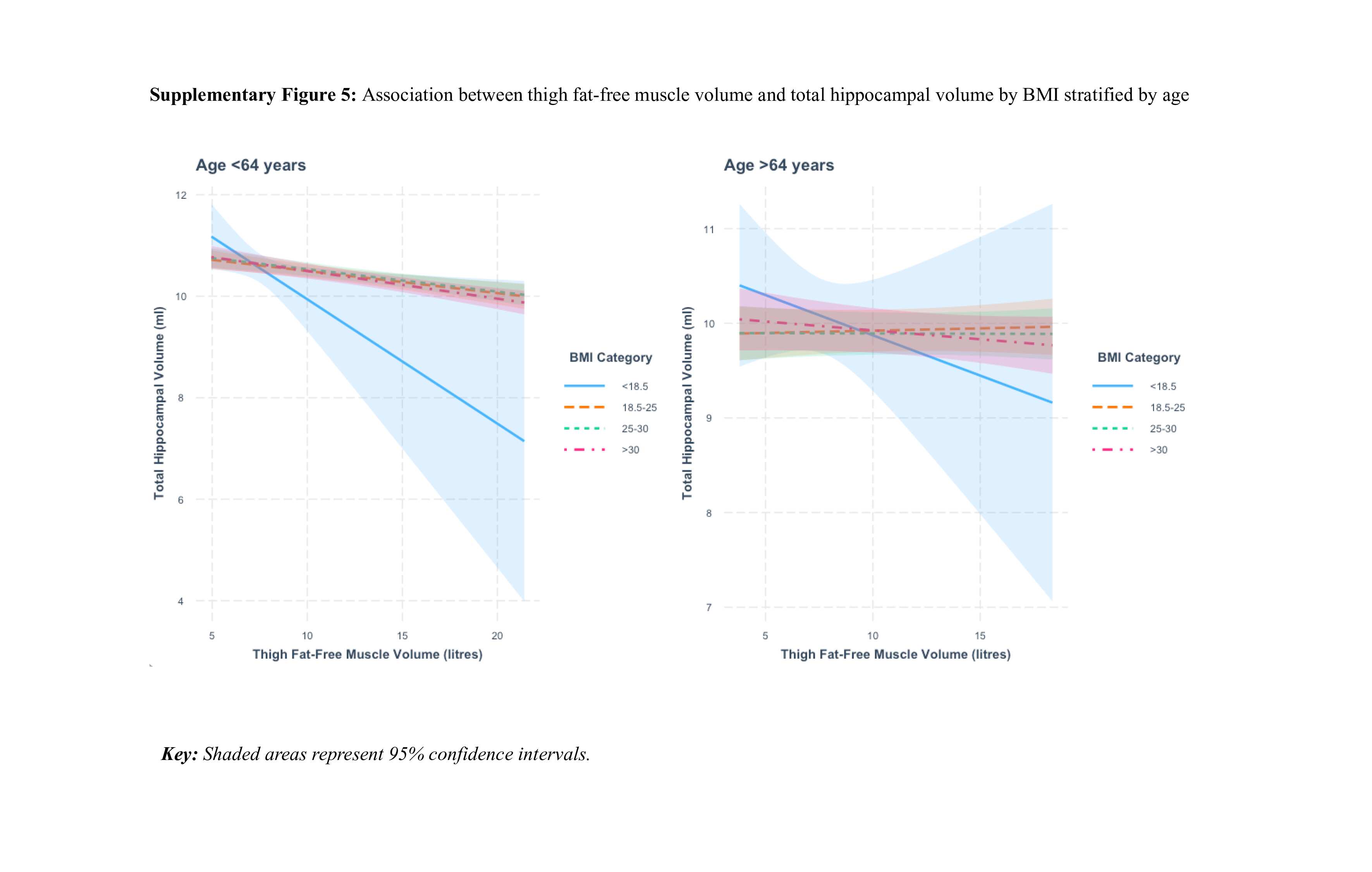

Supplement: Supplementary file 6 [file Image_5.JPEG]

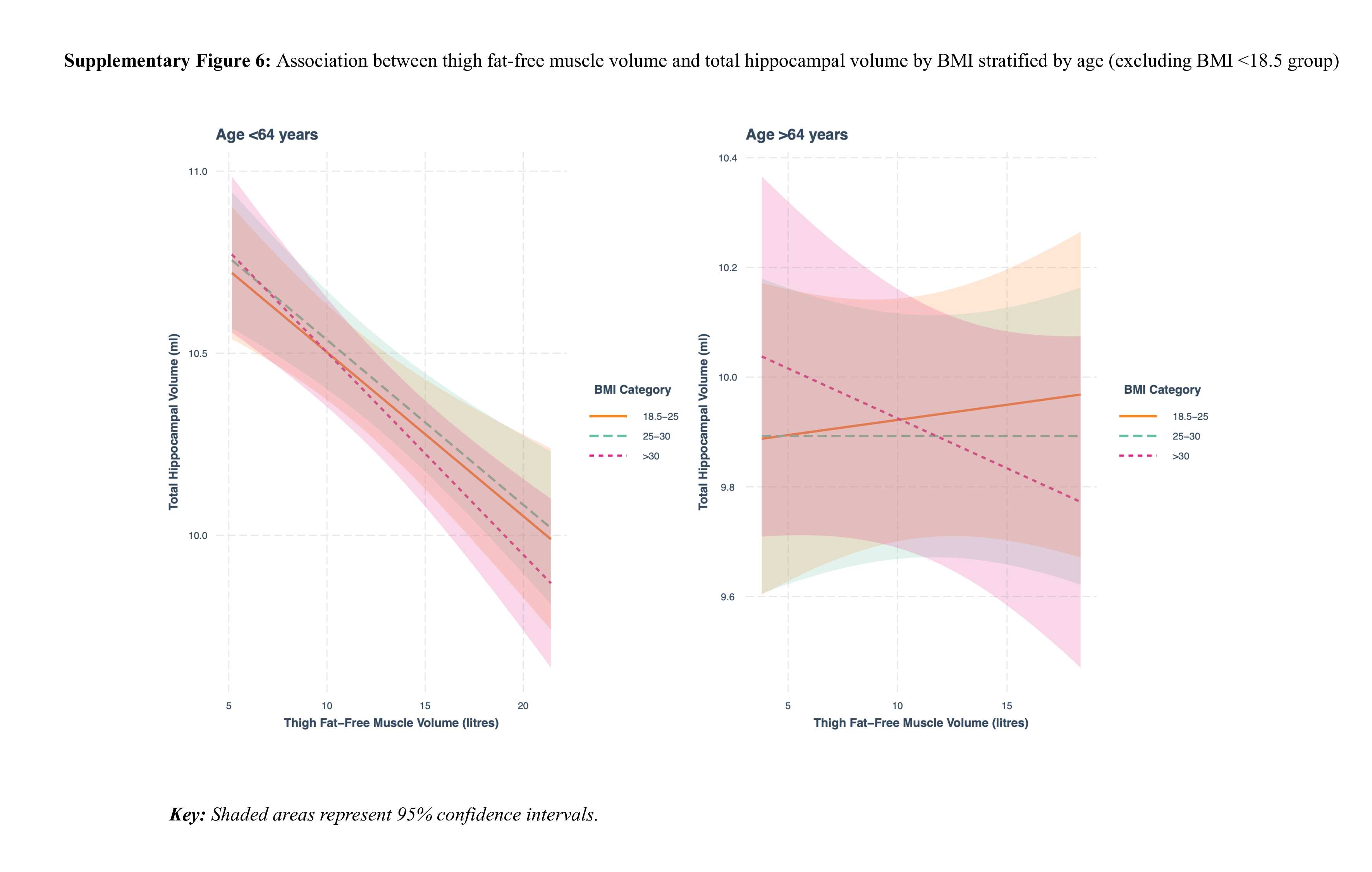

Supplement: Supplementary file 7 [file Image_6.JPEG]

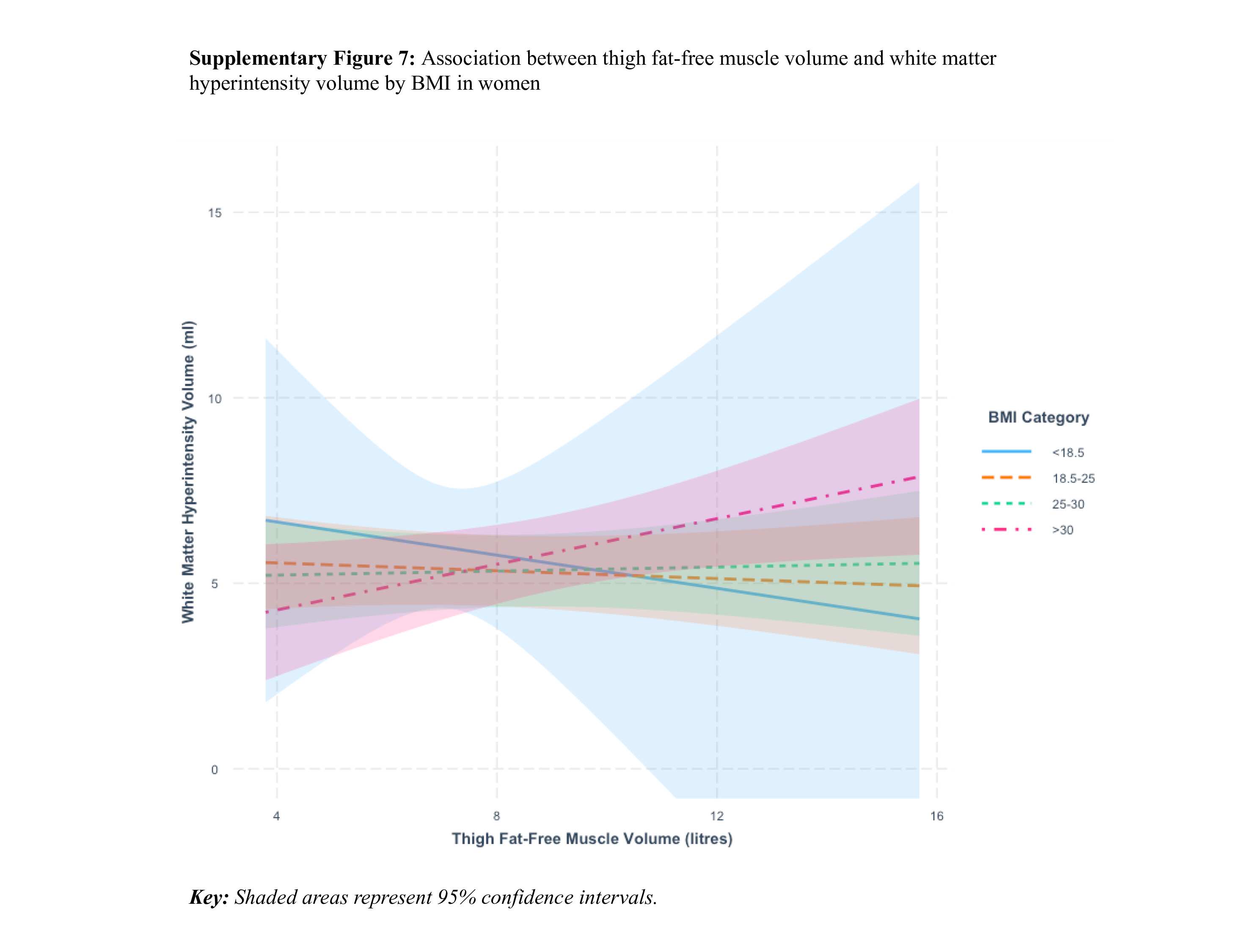

Supplement: Supplementary file 8 [file Image_7.JPEG]

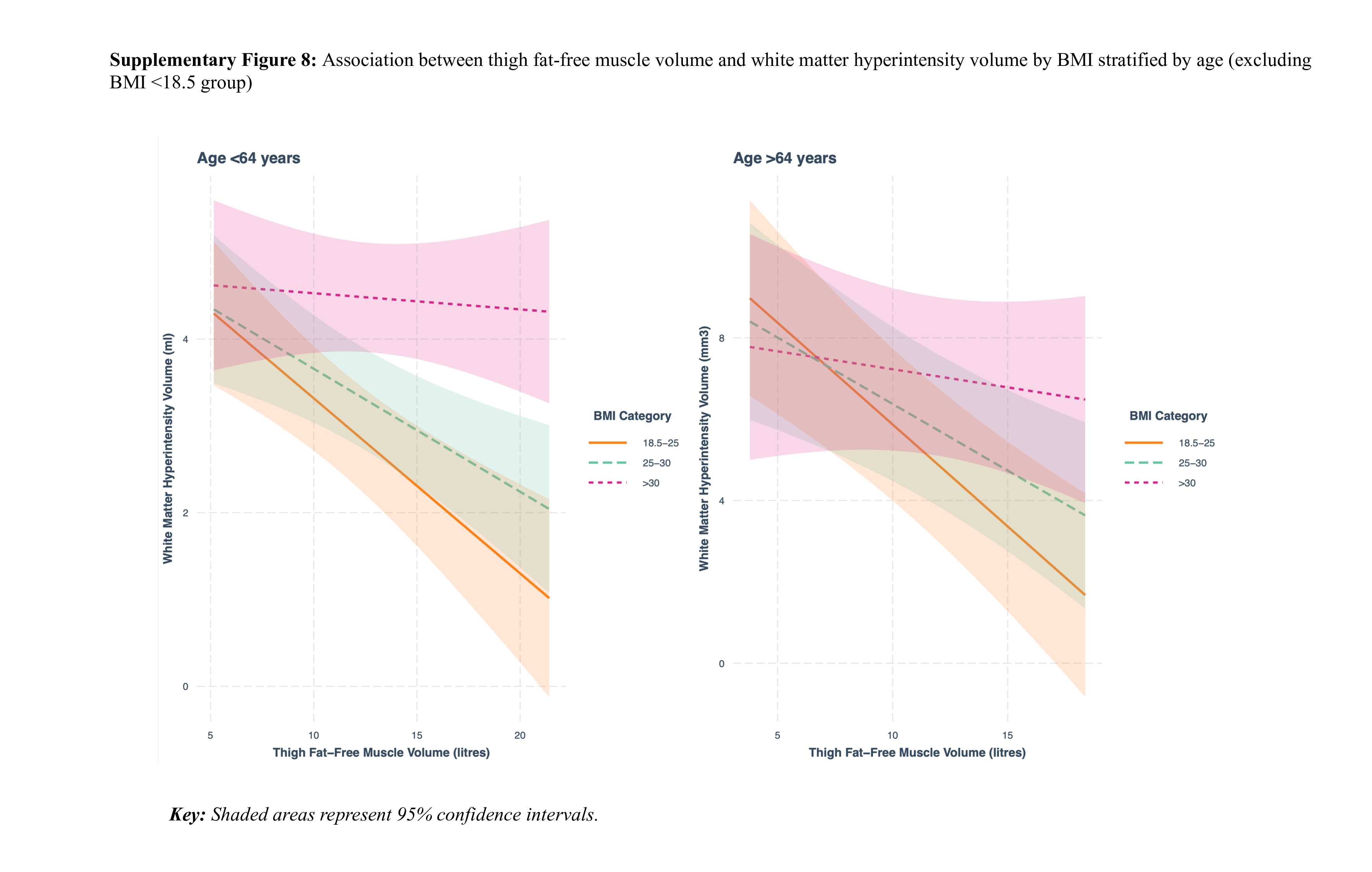

Supplement: Supplementary file 9 [file Image_8.JPEG]
